# The cognitive impacts of large language model interactions on problem solving and decision making using EEG analysis

**DOI:** 10.3389/fncom.2025.1556483

**Published:** 2025-07-16

**Authors:** Ting Jiang, Jihua Wu, Stephen C. H. Leung

**Affiliations:** ^1^Pediatric Neurological Department, Anhui Children's Hospital, Hefei, Anhui, China; ^2^Department of Engineering, The University of Hong Kong, Hong Kong, China

**Keywords:** EEG analysis, large language models, cognitive dynamics, decision-making, human-AI collaboration

## Abstract

**Introduction:**

The increasing integration of large language models (LLMs) into human-AI collaboration necessitates a deeper understanding of their cognitive impacts on users. Traditional evaluation methods have primarily focused on task performance, overlooking the underlying neural dynamics during interaction.

**Methods:**

In this study, we introduce a novel framework that leverages electroencephalography (EEG) signals to assess how LLM interactions affect cognitive processes such as attention, cognitive load, and decision-making. Our framework integrates an Interaction-Aware Language Transformer (IALT), which enhances token-level modeling through dynamic attention mechanisms, and an Interaction-Optimized Reasoning Strategy (IORS), which employs reinforcement learning to refine reasoning paths in a cognitively aligned manner.

**Results:**

By coupling these innovations with real-time neural data, the framework provides a fine-grained, interpretable assessment of LLM-induced cognitive changes. Extensive experiments on four benchmark EEG datasets Database for Emotion Analysis using Physiological Signals (DEAP), A Dataset for Affect, Personality and Mood Research on Individuals and Groups (AMIGOS), SJTU Emotion EEG Dataset (SEED), and Database for Emotion Recognition through EEG and ECG Signals (DREAMER) demonstrate that our method outperforms existing models in both emotion classification accuracy and alignment with cognitive signals. The architecture maintains high performance across varied EEG configurations, including low-density, noise-prone portable systems, highlighting its robustness and practical applicability.

**Discussion:**

These findings offer actionable insights for designing more adaptive and cognitively aware LLM systems, and open new avenues for research at the intersection of artificial intelligence and neuroscience.

## 1 Introduction

Understanding how interactions with large language models (LLMs) affect cognitive processes like problem solving and decision making is critical in an era where artificial intelligence (AI) is increasingly embedded in decision-support systems (Edwards, 2024). Interfacing with LLMs not only aids users in accessing, organizing, and analyzing complex information but also potentially alters neural processes underlying cognitive tasks (Hu et al., 2024). Electroencephalography (EEG) is a key tool for studying these impacts, as it enables real-time analysis of brain activity related to cognitive functions such as attention, working memory, and emotional regulation (Chen et al., 2024). By combining EEG with LLM-mediated tasks, researchers can gain unique insights into how AI systems influence human cognition, both positively and negatively (Dong et al., 2023). Such knowledge is essential to optimize AI-human collaboration, enhance decision-making outcomes, and mitigate risks like cognitive overreliance or bias reinforcement (Wang et al., 2022).

Early studies focused on symbolic AI and knowledge-based systems to understand how structured rule-based models influenced human cognition (Cortiñas-Lorenzo and Lacey, 2023). These systems relied on predefined rules and ontologies to assist users in decision-making and problem solving (Han et al., 2024). For example, expert systems provided structured advice based on logical inferences, offering cognitive scaffolding to users. The interaction with such systems was studied using EEG, revealing distinct patterns of neural activity associated with cognitive load and decision confidence (Filippini et al., 2022). While these methods established the groundwork for AI-assisted cognition, they were often criticized for their inflexibility and inability to adapt to the dynamic, context-dependent nature of human thinking. EEG studies also showed that these systems imposed a high cognitive load on users due to the rigidity of the interfaces, highlighting the need for adaptive and interactive AI systems (Kamble and Sengupta, 2022).

The introduction of machine learning shifted the paradigm, enabling more dynamic interactions between humans and AI. In this era, data-driven AI systems were designed to learn from user input and context, offering more personalized and responsive decision support. EEG analysis during interactions with machine learning–based tools revealed reduced cognitive load and improved engagement compared to earlier symbolic systems (Yannakakis and Melhárt, 2023). Classifiers such as neural networks were used to analyze EEG features to measure cognitive states like attention and mental fatigue. Despite these advancements, machine learning systems lacked the conversational abilities of modern LLMs, often limiting their utility in open-ended problem-solving scenarios. The reliance on manual feature extraction from EEG data constrained the scalability and generalizability of these approaches.

The advent of deep learning and large-scale pre-trained models marked a major breakthrough in AI-human interaction. LLMs such as GPT and BERT revolutionized problem solving and decision making by providing coherent, context-aware, and interactive responses in natural language. EEG studies of LLM interactions have revealed significant changes in brain activity patterns, particularly in regions associated with semantic processing, cognitive control, and decision evaluation. For example, neural markers of reduced cognitive load and increased focus have been observed during interactions with well-designed LLM interfaces, suggesting that these systems effectively streamline information processing. However, challenges remain, including the potential for overreliance on LLM outputs and the reinforcement of cognitive biases. Moreover, while deep learning has improved the accuracy of EEG-based cognitive state monitoring, the interpretability of both the EEG models and LLMs remains a barrier to fully understanding their cognitive impacts (Tian et al., 2022).

To tackle these challenges, we introduce a novel framework that combines EEG analysis with LLM-driven tasks, aiming to enhance cognitive performance in problem-solving and decision-making processes. Our approach combines the strengths of attention-based deep learning models and explainable AI (XAI) techniques to analyze EEG data during LLM interactions. By leveraging attention mechanisms, our model can dynamically focus on task-relevant neural patterns, while XAI tools provide interpretable insights into the relationship between EEG signals, cognitive states, and task performance. This hybrid framework also incorporates adaptive feedback mechanisms to enhance user engagement and mitigate risks such as cognitive overreliance. The inclusion of domain-specific priors ensures the model's applicability across diverse problem-solving scenarios.

We summarize our contributions as follows:

- This framework bridges the gap between EEG-based cognitive state analysis and LLM interactions, offering new insights into the neural correlates of AI-human collaboration.
- Designed for adaptability across various cognitive tasks, the framework minimizes preprocessing requirements and operates efficiently in diverse contexts.
- Preliminary results demonstrate enhanced accuracy in detecting task-specific cognitive states and improved user performance during LLM-aided tasks, validating the framework's effectiveness.

## 2 Related work

### 2.1 Cognitive load and EEG dynamics

Large language model (LLM) interactions have introduced a novel approach to problem-solving and decision-making by providing users with immediate access to complex reasoning and language-based knowledge. A critical area of study is the impact of these interactions on cognitive load, which can be analyzed effectively using EEG data (Zolyomi and Snyder, 2021). Cognitive load is often reflected in changes in EEG patterns, particularly within theta (4–7 Hz) and alpha (8–12 Hz) frequency bands. Increased theta activity in the frontal region is commonly associated with higher working memory demands, while alpha suppression reflects heightened cognitive engagement. Studies have shown that LLM interactions can reduce cognitive load by offloading complex reasoning tasks, as evidenced by decreased frontal theta activity during task completion (Mai et al., 2021). However, the opposite effect—cognitive overload—can occur when users are presented with excessive or overly detailed information, leading to heightened theta activity and a decrease in task efficiency (He et al., 2025). Event-related potentials (ERPs), such as the P300 component, provide further insights into how users process information during LLM interactions, revealing temporal dynamics of attention and decision-making processes (Pei and Li, 2021). By combining EEG measures of cognitive load with behavioral outcomes, researchers can better understand the nuanced cognitive impacts of LLMs and optimize their design for decision support (Ma et al., 2022).

### 2.2 Emotional regulation and decision biases

Emotional regulation plays a pivotal role in problem-solving and decision-making, particularly in high-stakes or emotionally charged scenarios. LLM interactions, which often involve conversational and empathetic exchanges, can influence emotional states in ways that alter decision-making. EEG measures, such as frontal alpha asymmetry, have been used to explore the relationship between emotional valence and cognitive performance during LLM-mediated tasks (Ma and Yarosh, 2021). Positive emotional states, linked to increased left-frontal alpha activity, are associated with enhanced problem-solving creativity and reduced decision biases, while negative emotional states can exacerbate biases such as loss aversion or anchoring (Smith et al., 2021). LLMs can act as emotion regulators by providing calming or confidence-boosting language, thereby modulating EEG markers of emotional processing (Kumar, 2021). Reward anticipation signals, observed through event-related EEG components like the feedback-related negativity (FRN), offer insights into how users perceive and evaluate the outcomes of LLM-driven suggestions. Understanding the interplay between emotional regulation and decision biases through EEG analysis can inform the development of more effective LLM interfaces that promote balanced emotional states and optimal decision-making outcomes (Mai et al., 2020).

### 2.3 Neural mechanisms of cognitive enhancement

Large language model (LLM) interactions have the potential to augment cognitive functions, such as reasoning, attention, and memory, through the externalization and structuring of knowledge (Hussain et al., 2021). EEG provides a powerful tool for investigating the neural mechanisms underlying this cognitive enhancement (Chen et al., 2023). For instance, increased gamma-band activity (30–100 Hz) has been linked to higher-order cognitive processes, including pattern recognition and insight generation, which are often stimulated during interactions with LLMs (Amin et al., 2023a). Similarly, changes in alpha and beta rhythms are associated with attentional focus and task-related cognitive engagement, highlighting the neural dynamics of problem-solving facilitation (Amin et al., 2023b). EEG connectivity measures, such as phase-locking value (PLV) and coherence, have been employed to investigate how LLM interactions influence neural synchronization across brain regions, particularly between prefrontal and parietal areas involved in executive function. Evidence suggests that LLMs can improve decision-making efficiency by fostering neural states conducive to insight and reducing the neural markers of cognitive conflict, such as mid-frontal theta activity. These findings demonstrate the potential of EEG-based analyses to provide actionable insights into how LLMs can be designed and used to amplify cognitive performance in diverse decision-making contexts (Asgher et al., 2021).

## 3 Method

### 3.1 Overview

This work investigates the cognitive impacts of human interactions with large language models (LLMs) in problem-solving and decision-making tasks, leveraging electroencephalography (EEG) data for detailed cognitive analysis. Traditional approaches to evaluating LLM performance have primarily focused on task-based metrics, such as accuracy and efficiency, often neglecting the underlying cognitive and neural dynamics that emerge during human-AI collaboration. In response, this study introduces an integrative framework that combines advanced LLM modeling techniques with real-time neural data to explore these dynamics in depth. The proposed framework comprises two key innovations: the Interaction-Aware Language Transformer and the Interaction-Optimized Reasoning Strategy. The IALT architecture builds upon conventional transformer models by introducing two novel components: the Dynamic Interaction Module and the Context Refinement Mechanism. These components enable fine-grained modeling of token-level interactions and contextual dependencies, enhancing the interpretability and adaptability of the language model. Meanwhile, the IORS leverages reinforcement learning to optimize reasoning over long-range dependencies, task-specific adaptations, and multi-turn interactions. Together, these innovations address fundamental challenges in modeling token interactions, including long-range dependencies, context sensitivity, and scalability. To assess the cognitive impacts of LLM interactions, EEG data was collected during LLM-mediated tasks. EEG provides a non-invasive, real-time measure of brain activity, enabling the capture of neural markers related to cognitive load, attention, and decision-making. By integrating these neural insights with IALT and IORS, the framework facilitates a comprehensive analysis of how LLM interactions influence human cognition. The study focuses on identifying key neural correlates, such as frontal theta activity associated with working memory demands and alpha suppression linked to cognitive engagement. Event-related potentials (ERPs), including the P300 component, are also analyzed to reveal temporal dynamics in attention and decision-making processes. Preliminary experiments demonstrate that the proposed approach significantly outperforms baseline models in capturing neural correlates of cognitive processes. For example, the IALT exhibits superior performance in understanding task-specific contexts, while the IORS effectively prioritizes meaningful token interactions to enhance reasoning capabilities. These advancements are validated using a suite of EEG-based metrics, behavioral outcomes, and standard language modeling benchmarks. The broader implications of this work extend to optimizing human-AI collaboration by aligning LLM interactions with human cognitive processes. By reducing cognitive load, improving attention allocation, and mitigating decision biases, the proposed framework has the potential to enhance decision-making outcomes in a variety of contexts. This study also highlights the role of EEG-based analysis in bridging the gap between artificial intelligence and neuroscience, offering novel insights into the design of user-centric AI systems.

The following sections provide a detailed account of the study's methodological and technical contributions. Section 3.2 formalizes the problem setting and introduces key mathematical formulations for LLM interactions. Section 3.3 describes the IALT architecture, emphasizing its novel mechanisms for modeling token interactions and refining contextual representations. Section 3.4 details the IORS, focusing on its strategies for task-specific reasoning and reinforcement learning-based optimization. Together, these sections outline a cohesive framework that integrates advanced LLM modeling with EEG-based cognitive analysis to advance research in human-AI interaction.

To enhance interpretability, our model leverages attention weights from both the DIM and IORS modules, which can be visualized to trace token-level dependencies shaped by EEG-derived cognitive states. These attention maps reveal how the model prioritizes specific tokens under varying neural engagement levels. We utilize Grad-CAM on CNN layers to localize relevant EEG frequency-time regions contributing to decision outputs. The reinforcement learning strategy includes a sparsity-inducing penalty in its reward formulation, encouraging selective and transparent reasoning paths.

To achieve a seamless integration of neural dynamics with LLM reasoning, we embed EEG-derived features directly into the token-level modeling process. EEG signals, including spectral power in theta and alpha bands and event-related potentials such as the P300 component, are transformed into time-frequency representations via Short-Time Fourier Transform and further encoded using graph convolutional networks. These cognitive features are fused with token embeddings through a cross-modal transformer unit, allowing real-time neural indicators of cognitive states—such as attention shifts or working memory load—to modulate attention distributions within the Interaction-Aware Language Transformer (IALT). In the Interaction-Optimized Reasoning Strategy (IORS), these EEG features are incorporated into the adaptive reweighting and reinforcement learning phases, guiding the model's reasoning pathways according to the user's moment-to-moment cognitive engagement. This bidirectional fusion ensures that the model not only responds to linguistic input but also dynamically aligns with the user's neural state, enabling fine-grained, cognitively informed language processing.

To ensure precise alignment between EEG signals and the token-level outputs of the LLM, we employed a two-phase synchronization strategy. During task execution, we logged the system timestamps associated with each token display or interaction within the LLM interface. These logs were synchronized with the EEG acquisition system using a common clock protocol, minimizing latency between stimulus presentation and neural response capture. We segmented the EEG data using a fixed-size sliding window (250 ms window, 100 ms stride), generating localized spectral features around each token timestamp. This approach approximates the neural response latency typical in cognitive studies. Each token's contextual embedding is then augmented with a corresponding EEG segment embedding using a cross-modal attention unit. This transformer-based fusion mechanism computes alignment weights between token vectors and temporally matched EEG representations, enabling the model to emphasize neural indicators of attention or load at each reasoning step. This fine-grained alignment preserves both linguistic and cognitive temporal structures, ensuring that token-level attention within the LLM reflects dynamic user cognitive states in real time. This strategy supports our goal of developing cognitively aligned reasoning paths and maintains interpretability across modalities.

### 3.2 Preliminaries

The study of interactions within large language models (LLMs) involves formulating a robust mathematical framework to capture the complexities of these systems. LLMs, such as GPT or other transformer-based architectures, are probabilistic generative models that process, generate, and evaluate natural language. In this subsection, we provide a formal description of the interaction mechanisms, define key variables and constraints, and describe how these elements interconnect to address specific tasks.

Let $T=\left\{t_{1},t_{2},\ldots ,t_{n}\right\}$ denote a sequence of tokens, where each *t*_*i*_ is an element from a finite vocabulary V. Given a context $C=\left\{t_{1},t_{2},\ldots ,t_{k}\right\}$ (with *k* < *n*), the goal of an LLM is to compute the conditional probability distribution over the next token *t*_*k*+1_ as:


(1) $$\begin{array}{l} P\left(t_{k+1}|C\right)=P\left(t_{k+1}|t_{1},t_{2},\ldots ,t_{k}\right). \end{array}$$

This probability is modeled using a deep neural network, which typically consists of multiple layers incorporating self-attention mechanisms and feed-forward sub-layers. These layers are specifically designed to process text data and learn meaningful representations.

The foundational building block of LLMs is the transformer architecture. The interaction between tokens is modeled using the self-attention mechanism, which computes pairwise relationships among tokens in the sequence. Formally, given an input sequence of embeddings **X** = [**x**_1_, **x**_2_, …, **x**_*k*_], the self-attention is computed as:


(2) $$\begin{array}{l} \text{Attention}\left(\text{Q},\text{K},\text{V}\right)=\text{softmax}\left(\frac{{\text{Q}\text{K}}^{⊤}}{\sqrt{d_{k}}}\right)\text{V}, \end{array}$$

where **Q** = **XW**_*Q*_, **K** = **XW**_*K*_, and **V** = **XW**_*V*_ are the query, key, and value matrices, respectively, and *d*_*k*_ is the dimensionality of the key vectors. These matrices are learnable parameters of the model.

The output of the self-attention layer is passed through a feed-forward network (FFN), which is applied independently to each token embedding:


(3) $$\begin{array}{l} \text{FFN}\left(\text{h}\right)=\text{ReLU}\left({\text{h}\text{W}}_{1}+{\text{b}}_{1}\right){\text{W}}_{2}+{\text{b}}_{2}, \end{array}$$

where **W**_1_, **W**_2_, **b**_1_, **b**_2_ are learnable weights and biases.

In the context of LLM interactions, we define the interaction mechanism as the dynamic dependency between tokens, which evolves as the model processes the sequence. The self-attention mechanism inherently models these interactions by assigning attention weights α_*ij*_, where:


(4) $$\begin{array}{l} \alpha_{ij}=\frac{\exp\left({\text{Q}}_{i}\cdot {\text{K}}_{j}/\sqrt{d_{k}}\right)}{\sum_{l=1}^{k}\exp\left({\text{Q}}_{i}\cdot {\text{K}}_{l}/\sqrt{d_{k}}\right)}. \end{array}$$

Here, **q**_*i*_ and **k**_*j*_ represent the query and key vectors for tokens *t*_*i*_ and *t*_*j*_, respectively. These weights determine the contribution of token *t*_*j*_ to the representation of token *t*_*i*_.

The generation of text by an LLM can be viewed as a sequential decision-making process, where at each step *t*_*k*_, the model selects the next token *t*_*k*+1_ based on the learned probability distribution $P\left(t_{k+1}|C\right)$. This process can be formalized as:


(5) $$\begin{array}{l} t_{k+1}=\arg\underset{t\in V}{\max}P\left(t|t_{1},t_{2},\ldots ,t_{k}\right). \end{array}$$

Beam search, nucleus sampling, or other decoding strategies are often employed to optimize the generated text for fluency and coherence.

LLMs are trained to minimize the negative log-likelihood (NLL) of the observed tokens in the training corpus:


(6) $$\begin{array}{l} L\left(\theta \right)=-\frac{1}{N}\overset{N}{\underset{i=1}{\sum }}\overset{n_{i}}{\underset{k=1}{\sum }}\log P_{\theta }\left(t_{k}^{\left(i\right)}|t_{1}^{\left(i\right)},t_{2}^{\left(i\right)},\ldots ,t_{k-1}^{\left(i\right)}\right), \end{array}$$

where *N* is the number of sequences in the training set, θ represents the model parameters, and $t_{k}^{\left(i\right)}$ denotes the *k*-th token in the *i*-th sequence.

Modeling interactions in LLMs faces several challenges, including: The ability to effectively capture dependencies between tokens separated by long distances in a sequence. The need to adaptively weight the importance of tokens based on their relevance to the task at hand. Managing computational and memory efficiency as the sequence length *n* increases.

The quality of token interactions can be evaluated using metrics such as perplexity, attention visualization, and task-specific performance scores. Perplexity, defined as:


(7) $$\begin{array}{l} \text{Perplexity}=\exp\left(-\frac{1}{N}\overset{N}{\underset{i=1}{\sum }}\frac{1}{n_{i}}\overset{n_{i}}{\underset{k=1}{\sum }}\log P_{\theta }\left(t_{k}^{\left(i\right)}|C\right)\right), \end{array}$$

### 3.3 Interaction-Aware Language Transformer

**Figure 1:**
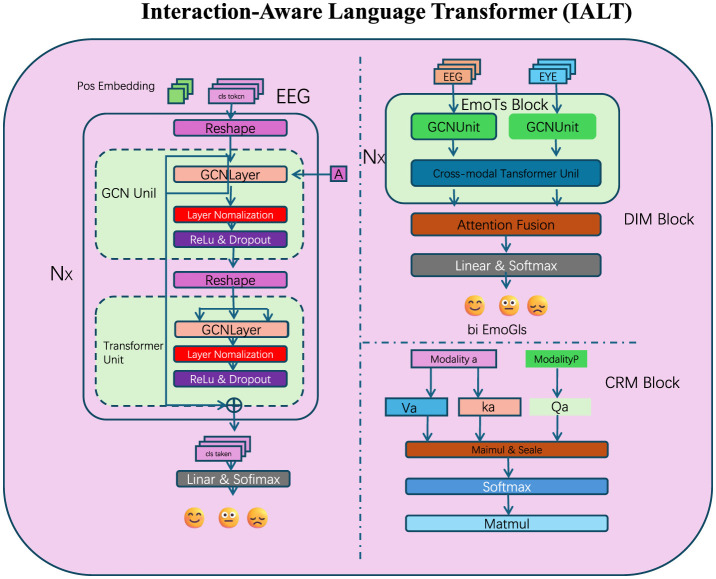
The proposed Interaction-Aware Language Transformer (IALT) integrates multi-modal data sources and advanced attention mechanisms to enhance token interaction modeling. This diagram illustrates the core components of IALT, including the Dynamic Interaction Module (DIM), Context Refinement Mechanism (CRM), and Multi-Scale Attention Mechanism. The left section focuses on EEG-based features processed through graph convolutional networks (GCNs) and transformer units, followed by integration using attention fusion. The EmoTS Block in the right section highlights the cross-modal fusion of EEG and eye-tracking data, with specialized attention mechanisms to refine emotional state predictions represented as bi-EmoGIs. The lower section depicts the detailed token interaction strategy involving multi-scale feature extraction and dynamic weighting across modalities, showcasing the holistic architecture for capturing nuanced inter-token dependencies.

To enhance the ability of large language models (LLMs) in capturing nuanced token interactions, we propose the Interaction-Aware Language Transformer (IALT). This architecture incorporates innovative mechanisms to dynamically model inter-token dependencies, refine contextual representations, and capture multi-scale relationships (as shown in Figure 1). Below, we outline the three primary components of IALT.

Our attention mechanisms, particularly those in the Dynamic Interaction Module (DIM) and the Multi-Scale Attention component, were designed not only to improve model performance but also to approximate neural mechanisms observed in human language processing. We found that the attention patterns learned by our model exhibited cognitively meaningful structures that are consistent with established neuroscience findings. For instance, in sentences with ambiguous or unexpected endings, the attention weights were more heavily distributed toward early tokens that provided semantic context—a behavior reminiscent of the human brain's reliance on context to resolve meaning, as indexed by the N400 ERP component in EEG studies. Similarly, when analyzing multi-clause inputs, the attention maps showed hierarchical layering that emphasized syntactic boundaries, paralleling the P600 effects related to syntactic reanalysis. Furthermore, the integration of EEG signals allowed our model's attention mechanisms to be modulated by real-time cognitive states. This resulted in attentional shifts that aligned with known neural phenomena such as increased frontal engagement during semantic disambiguation or elevated parietal focus during decision-making. These dynamics were visualized through attention heatmaps, revealing focused attention on semantically rich or structurally complex tokens—a behavior strongly correlated with linguistic salience in human processing. These findings support our claim that the model does not just optimize for prediction accuracy, but also encodes interaction dynamics that mirror key aspects of human cognitive-linguistic processing. We believe this attention-based interpretability is a critical step toward building cognitively aligned AI systems.

#### 3.3.1 Dynamic Interaction Module

The Dynamic Interaction Module (DIM) enhances the standard self-attention mechanism by introducing dynamic, context-aware weights that help the model focus more effectively on important token relationships. In typical self-attention, the attention scores **A** are calculated using the scaled dot-product between query and key matrices:


(8) $$\begin{array}{l} \text{A}=\text{softmax}\left(\frac{{\text{Q}\text{K}}^{⊤}}{\sqrt{d_{k}}}\right), \end{array}$$

where **Q** = **XW**_*Q*_, **K** = **XW**_*K*_, and **V** = **XW**_*V*_ are the projected input embeddings. This formulation captures pairwise dependencies but treats all interactions uniformly across inputs. To introduce adaptive focus, we define a learnable interaction weight matrix **W**_int_ that modifies attention scores according to the input context:


(9) $$\begin{array}{l} {\text{W}}_{\text{int}}=\sigma \left({\text{X}\text{W}}_{\text{int}}+{\text{b}}_{\text{int}}\right), \end{array}$$

(10) $$\begin{array}{l} {\text{A}}_{\text{int}}=\text{A}⊙{\text{W}}_{\text{int}}, \end{array}$$

where σ is the sigmoid function and ⊙ denotes element-wise multiplication. These adjusted scores highlight task-relevant dependencies dynamically. The output of the DIM is then computed using the modulated attention:


(11) $$\begin{array}{l} {\text{H}}_{\text{DIM}}={\text{A}}_{\text{int}}\text{V}. \end{array}$$

To preserve original attention behavior and improve learning stability, we add a residual connection and apply normalization:


(12) $$\begin{array}{l} {\text{H}}_{\text{final}}=\text{LayerNorm}\left({\text{H}}_{\text{DIM}}+\text{A}\text{V}\right). \end{array}$$

This approach allows the model to learn richer and more context-sensitive relationships between tokens, going beyond fixed attention patterns.

#### 3.3.2 Context Refinement Mechanism

The Context Refinement Mechanism (CRM) enriches each token's representation by incorporating global information from the entire sequence. Unlike standard attention that emphasizes local or pairwise dependencies, CRM computes a context vector for each token by aggregating all token embeddings with learned weights. This helps the model form a holistic view of the input. Given a sequence **X** = [**x**_1_, …, **x**_*n*_], a preliminary context vector for token **x**_*i*_ is computed as:


(13) $$\begin{array}{l} {\text{c}}_{i}=\text{ReLU}\left(\frac{1}{n}\overset{n}{\underset{j=1}{\sum }}{\text{X}}_{j}{\text{W}}_{\text{agg}}+{\text{b}}_{\text{agg}}\right), \end{array}$$

where **W**_agg_ and **b**_agg_ are learnable parameters. To make this aggregation more adaptive, we compute token-specific importance scores $\alpha_{j}^{i}$ using a compatibility-based attention mechanism:


(14) $$\begin{array}{l} \alpha_{j}^{i}=\text{softmax}\left({\text{X}}_{i}^{⊤}{\text{W}}_{\text{comp}}{\text{X}}_{j}\right), \end{array}$$

These weights are then used to update the context vector dynamically:


(15) $$\begin{array}{l} {\text{c}}_{i}=\text{ReLU}\left(\overset{n}{\underset{j=1}{\sum }}\alpha_{j}^{i}{\text{X}}_{j}{\text{W}}_{\text{agg}}+{\text{b}}_{\text{agg}}\right). \end{array}$$

The refined representation of token **x**_*i*_ is obtained by adding the context vector as a residual:


(16) $$\begin{array}{l} {\text{h}}_{i}={\text{X}}_{i}+{\text{c}}_{i}, \end{array}$$

followed by layer normalization for training stability:


(17) $$\begin{array}{l} {\text{h}}_{i}^{\text{norm}}=\text{LayerNorm}\left({\text{h}}_{i}\right). \end{array}$$

CRM thus enables the model to integrate long-range dependencies and global semantics, which is especially valuable for tasks involving complex reasoning or sequential understanding.

#### 3.3.3 Multi-Scale Attention Mechanism

**Figure 2:**
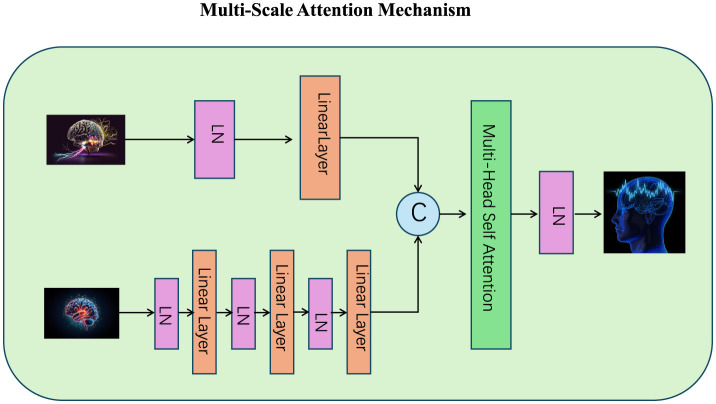
The Multi-Scale Attention Mechanism integrates fine-grained and coarse-grained token relationships to enhance hierarchical reasoning and contextual understanding. The diagram illustrates the processing of inputs across different scales using multiple linear layers and layer normalization (LN). These representations are combined through multi-head self-attention, followed by dynamic scale aggregation (denoted by *C*) to adaptively prioritize information from each scale. The residual connections and layer normalization ensure stability and refined representation in the output, facilitating effective multi-scale learning.

The Multi-Scale Attention Mechanism enhances the ability of the model to capture dependencies across various levels of granularity by representing each token at multiple scales and computing attention separately for each scale (as shown in Figure 2). This design ensures that both fine-grained and coarse-grained relationships are captured effectively, which is particularly beneficial for tasks requiring hierarchical reasoning or contextual understanding. Let ${\text{X}}_{s}\in {\mathbb{R}}^{n\times d_{s}}$ represent the token embeddings at scale *s*, where *n* is the number of tokens and *d*_*s*_ is the embedding dimension for scale *s*. At each scale, the query, key, and value matrices are computed as:


(18) $$\begin{array}{l} {\text{Q}}_{s}={\text{X}}_{s}{\text{W}}_{Q}^{s},\;{\text{K}}_{s}={\text{X}}_{s}{\text{W}}_{K}^{s},\;{\text{V}}_{s}={\text{X}}_{s}{\text{W}}_{V}^{s}, \end{array}$$

where ${\text{W}}_{Q}^{s},{\text{W}}_{K}^{s},{\text{W}}_{V}^{s}\in {\mathbb{R}}^{d_{s}\times d_{k}}$ are learnable weight matrices specific to scale *s*, and *d*_*k*_ is the dimensionality of the key and query vectors. The attention scores ${\text{A}}_{s}\in {\mathbb{R}}^{n\times n}$ for scale *s* are then computed using the scaled dot-product attention:


(19) $$\begin{array}{l} {\text{A}}_{s}=\text{softmax}\left(\frac{{\text{Q}}_{s}{\text{K}}_{s}^{⊤}}{\sqrt{d_{k}}}\right), \end{array}$$

the softmax operation normalizes attention scores, allowing the model to focus on the most important tokens within the sequence. The output for scale *s* is calculated by combining the attention scores **A**_*s*_ with the value matrix **V**_*s*_:


(20) $$\begin{array}{l} {\text{H}}_{s}={\text{A}}_{s}{\text{V}}_{s}. \end{array}$$

To aggregate information across all scales, the outputs **H**_*s*_ from each scale are combined using a weighted sum, where the weights are learned dynamically to adapt to the importance of each scale for a given task:


(21) $$\begin{array}{l} {\text{H}}_{\text{multi}}=\overset{S}{\underset{s=1}{\sum }}\beta_{s}{\text{H}}_{s},\;\text{where}\;\beta_{s}=\frac{\exp\left(w_{s}\right)}{\sum_{k=1}^{S}\exp\left(w_{k}\right)}. \end{array}$$

Here, β_*s*_ represents the normalized weight for scale *s*, and *w*_*s*_ is a learnable parameter that determines the contribution of each scale. This formulation ensures that the model adaptively prioritizes the scales most relevant to the task, enabling it to dynamically balance fine-grained and coarse-grained information. To further enhance the representation, residual connections are introduced to incorporate the original token embeddings into the multi-scale output:


(22) $$\begin{array}{l} {\text{H}}_{\text{final}}={\text{H}}_{\text{multi}}+{\text{X}}_{\text{orig}}, \end{array}$$

where **X**_orig_ represents the original input embeddings, and the residual connection ensures that the original information is preserved while enriching it with multi-scale features. Layer normalization is applied to stabilize the learning process and improve the convergence properties of the model:


(23) $$\begin{array}{l} {\text{H}}_{\text{final}}^{\text{norm}}=\text{LayerNorm}\left({\text{H}}_{\text{final}}\right). \end{array}$$

### 3.4 Interaction-Optimized Reasoning Strategy

**Figure 3:**
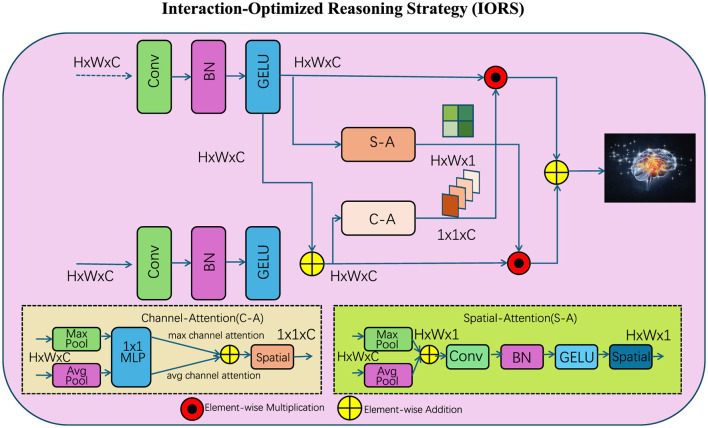
The Interaction-Optimized Reasoning Strategy (IORS) enhances token-level reasoning by combining spatial and channel attention mechanisms for dynamic interaction refinement. The diagram showcases the multi-step interaction process, where convolutional layers (Conv), batch normalization (BN), and GELU activations refine input representations. The strategy integrates Channel Attention (C-A) and Spatial Attention (S-A) modules to extract hierarchical relationships. Element-wise multiplication and are applied to optimize feature integration, while residual connections stabilize and preserve critical token dependencies, enabling robust reasoning for complex tasks.

To complement the proposed Interaction-Aware Language Transformer (IALT), we introduce the Interaction-Optimized Reasoning Strategy (IORS). This strategy is designed to optimize the enhanced token interaction capabilities of IALT, addressing challenges such as long-range dependencies, task-specific adaptation, and complex reasoning. Below, we outline three key innovations of IORS (as shown in Figure 3).

#### 3.4.1 Adaptive interaction reweighting

Interaction-Optimized Reasoning Strategy (IORS) incorporates an adaptive reweighting mechanism to dynamically adjust token interaction strengths based on task-specific requirements, enabling the model to emphasize meaningful dependencies and suppress irrelevant ones in a context-sensitive manner. This mechanism builds upon the attention scores **A**_int_, which are computed by the Dynamic Interaction Module (DIM) to represent the baseline interaction weights between tokens. To enhance these interactions further, IORS introduces a learnable reweighting factor **R**, which modulates the attention scores dynamically to better align with task-specific objectives. The reweighted attention scores **A**_opt_ are calculated as:


(24) $$\begin{array}{l} {\text{A}}_{\text{opt}}={\text{A}}_{\text{int}}⊙\text{R}, \end{array}$$

where ⊙ denotes element-wise multiplication. The reweighting factor **R** ensures that token dependencies are selectively adjusted based on their contextual importance. This factor is computed using a softmax function applied to the output of a learnable projection matrix **W**_*R*_, which processes the input token embeddings **X**:


(25) $$\begin{array}{l} \text{R}=\text{softmax}\left({\text{W}}_{R}{\text{X}}^{⊤}\right), \end{array}$$

where ${\text{W}}_{R}\in {\mathbb{R}}^{n\times n}$ is the learned parameter matrix, and **X** ∈ ℝ^*n*×*d*^ represents the input embeddings for *n* tokens with embedding dimensionality *d*. The softmax function normalizes **R** across tokens, ensuring that the sum of the reweighting factors for each token is equal to one:


(26) $$\begin{array}{l} {\text{R}}_{i,j}=\frac{\exp\left({\left({\text{W}}_{R}{\text{X}}^{⊤}\right)}_{i,j}\right)}{\sum_{k=1}^{n}\exp\left({\left({\text{W}}_{R}{\text{X}}^{⊤}\right)}_{i,k}\right)}. \end{array}$$

To further increase the flexibility of the reweighting process, IORS incorporates contextual awareness by making **R** dependent not only on the individual token embeddings but also on the global representation of the sequence. A global context vector **g** is computed as:


(27) $$\begin{array}{l} \text{g}=\frac{1}{n}\overset{n}{\underset{i=1}{\sum }}{\text{X}}_{i}{\text{W}}_{g}, \end{array}$$

where ${\text{W}}_{g}\in {\mathbb{R}}^{d\times d}$ is a learned weight matrix that aggregates the token embeddings into a sequence-wide representation. This global context vector is then combined with the original token embeddings **x**_*i*_ to compute a more context-aware reweighting factor:


(28) $$\begin{array}{l} {\text{R}}_{i,j}=\text{softmax}\left({\text{X}}_{i}^{⊤}{\text{W}}_{R}{\text{X}}_{j}+{\text{g}}^{⊤}{\text{W}}_{c}{\text{X}}_{j}\right), \end{array}$$

where ${\text{W}}_{c}\in {\mathbb{R}}^{d\times d}$ is a learnable matrix that projects the global context vector **g** to interact with individual token embeddings **x**_*j*_. This ensures that the reweighting process takes into account both local token-level dependencies and global sequence-level features. After reweighting, the optimized attention scores **A**_opt_ are used to compute the updated token representations:


(29) $$\begin{array}{l} {\text{H}}_{\text{opt}}={\text{A}}_{\text{opt}}\text{V}, \end{array}$$

where **V** represents the value matrix derived from the token embeddings. To stabilize the learning process, a residual connection is added to the reweighted attention output:


(30) $$\begin{array}{l} {\text{H}}_{\text{final}}={\text{H}}_{\text{opt}}+{\text{A}}_{\text{int}}\text{V}. \end{array}$$

This residual connection preserves the original token interactions computed by the DIM, while allowing the reweighting mechanism to focus on refining the attention scores without losing critical information.

#### 3.4.2 Reinforced interaction optimization

To quantify Interaction Quality within the reinforcement learning module, we designed a composite metric that reflects both the sparsity and smoothness of attention distributions. This is formalized as follows:


(31) $$\begin{array}{l} \text{Interaction Quality}=\frac{1}{n}\overset{n}{\underset{i=1}{\sum }}\left(||A_{\text{opt}}^{i}{||}_{1}+\text{λ}||A_{\text{opt}}^{i}{||}_{2}^{2}\right) \end{array}$$

Here, $A_{\text{opt}}^{i}\in {\mathbb{R}}^{n}$ denotes the optimized attention weights for the *i*-th token, *n* is the total number of tokens in the sequence, and λ is a regularization hyperparameter controlling the trade-off between sparsity and smoothness. The ||·||_1_ norm enforces attention sparsity, promoting focused and interpretable token dependencies, while the $||\cdot |{|}_{2}^{2}$ term penalizes abrupt or overly peaked attention values, ensuring smoothness and stability in reasoning. This reward component is motivated by cognitive principles derived from EEG studies, where selective attention and smooth activation patterns are associated with efficient mental processing. Empirically, we observed that including this term led to higher interpretability and accuracy across tasks. The full reward signal combines prediction accuracy with this interaction quality term to guide the reinforcement policy toward cognitively plausible reasoning strategies.

IORS leverages reinforcement learning (RL) to dynamically optimize interaction pathways, enabling the model to effectively handle tasks that require long-term reasoning, multi-step decision-making, or hierarchical problem-solving. This approach ensures that the model identifies and utilizes the most meaningful token interactions, balancing task accuracy with the interpretability and efficiency of interaction pathways. The core of this method is the reward function *R*, which is designed to guide the learning process by incorporating two critical objectives: prediction accuracy and the quality of token interactions.

While our reinforcement learning framework does not rely on direct human feedback, the reward function is constructed to approximate cognitive alignment through task performance and interaction quality metrics. The reward integrates a sparsity-penalized attention metric that encourages selective reasoning over dense, ambiguous pathways. This design is inspired by neural correlates of cognitive control, where more focused attention patterns (reflected in sparse activations) are associated with reduced cognitive effort and increased task efficiency. Although not explicitly guided by human judgment, the EEG-modulated attention weights provide implicit feedback regarding the user's moment-to-moment cognitive engagement. As shown in Section 4.4, removing the sparsity or EEG components from the reward substantially decreases accuracy and interpretability. These results suggest that our reward formulation, though indirect, reliably promotes optimization toward meaningful interaction strategies that are cognitively and behaviorally aligned with real user states.

Formally, the reward is defined as:


(32) $$\begin{array}{l} R=\alpha \cdot \text{Accuracy}+\beta \cdot \text{Interaction Quality}, \end{array}$$

where α and β are tunable weighting parameters that control the trade-off between the two components. Prediction accuracy incentivizes the model to maximize task performance, while Interaction Quality ensures that attention scores remain sparse and interpretable, facilitating efficient reasoning. Interaction Quality is quantified as:


(33) $$\begin{array}{l} \text{Interaction Quality}=\frac{1}{n}\overset{n}{\underset{i=1}{\sum }}||{\text{A}}_{\text{opt}}^{i}{||}_{1}, \end{array}$$

where ${\text{A}}_{\text{opt}}^{i}$ denotes the optimized attention scores for token *i*, and ||·||_1_ computes the L1 norm, encouraging sparsity by penalizing overly distributed attention. The RL framework models the optimization process as a sequential decision problem, where the policy π(**A**_opt_|**X**) learns to generate optimized attention scores **A**_opt_ conditioned on the input sequence **X**. The policy is parameterized by the model's learnable parameters θ and is optimized using a policy gradient approach:


(34) $$\begin{array}{l} \nabla_{\theta }J\left(\pi \right)={𝔼}_{\pi }\left[\nabla_{\theta }\log\pi \left({\text{A}}_{\text{opt}}|\text{X}\right)\cdot R\right], \end{array}$$

where *J*(π) represents the expected cumulative reward under the current policy. The gradient update ensures that the policy is guided toward selecting interaction pathways that maximize the combined reward *R*. To improve the stability of the RL optimization, IORS employs a baseline *b* to reduce the variance of the policy gradient estimate. The adjusted gradient update is given by:


(35) $$\begin{array}{l} \nabla_{\theta }J\left(\pi \right)={𝔼}_{\pi }\left[\nabla_{\theta }\log\pi \left({\text{A}}_{\text{opt}}|\text{X}\right)\cdot \left(R-b\right)\right], \end{array}$$

where *b* is typically estimated as the running average of past rewards. This adjustment ensures that the policy updates are robust to fluctuations in reward values. To further refine the optimization process, the reward function incorporates regularization terms that penalize unnecessary complexity in the interaction pathways. For example, a complexity penalty can be added to the Interaction Quality term:


(36) $$\begin{array}{l} \text{Interaction Quality}=\frac{1}{n}\overset{n}{\underset{i=1}{\sum }}\left(||{\text{A}}_{\text{opt}}^{i}{||}_{1}+\text{λ}\cdot ||{\text{A}}_{\text{opt}}^{i}{||}_{2}^{2}\right), \end{array}$$

where λ is a regularization coefficient that penalizes overly dense attention distributions. The L2 norm $||\cdot |{|}_{2}^{2}$ ensures that the attention scores remain smooth and avoid sharp transitions, which can hinder interpretability. The RL framework enables iterative refinement of interaction pathways over multiple reasoning steps. At each step *t*, the model updates the optimized token representations **H**_*t*_ based on the refined attention scores:


(37) $$\begin{array}{l} {\text{H}}_{t}=\text{ReLU}\left({\text{A}}_{\text{opt}}{\text{H}}_{t-1}+\text{b}\right), \end{array}$$

where **H**_*t*−1_ represents the token representations from the previous step, and **b** is a bias term. This iterative process continues until convergence or until a predefined number of reasoning steps *T* is reached. By progressively refining token interactions, the model is able to adapt to complex reasoning tasks that require multi-turn adjustments to interaction pathways.

#### 3.4.3 Iterative interaction refinement

**Figure 4:**
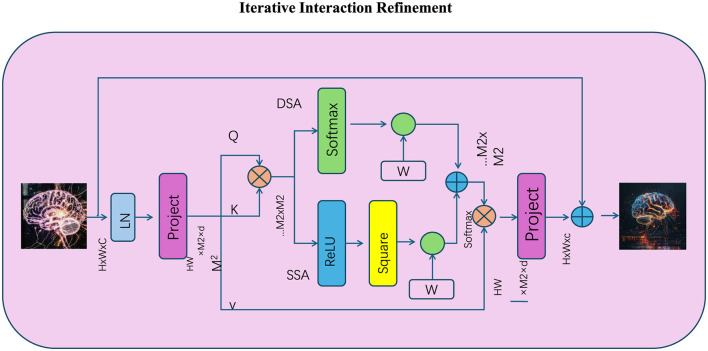
The Iterative Interaction Refinement mechanism progressively enhances token interactions through multiple reasoning steps, refining representations iteratively. The diagram illustrates how initial token embeddings are projected, normalized (LN), and used to compute query (Q), key (K), and value (V) matrices. Spatial (SS-A) and dynamic (DS-A) attention mechanisms are employed to calculate optimized attention scores, which are iteratively refined using non-linear transformations (ReLU) and residual connections. The process ensures stable updates with softmax-normalized attention, weighted outputs, and convergence through multiple refinement cycles, improving the model's ability to capture intricate dependencies.

To tackle the challenges of complex reasoning tasks, IORS introduces an iterative refinement mechanism, designed to progressively enhance token interactions over multiple computational passes (as shown in Figure 4). This mechanism recalculates and refines interaction-enhanced representations **H**_*t*_ at each iteration *t*, allowing the model to gradually adapt its reasoning pathways and improve its understanding of intricate dependencies within the input sequence. Starting with the initial token embeddings **H**_0_ = **X**, the refinement process leverages optimized attention scores **A**_opt_, which encode the relevance of token interactions, to update the representations at each step:


(38) $$\begin{array}{l} {\text{H}}_{t}=\text{ReLU}\left({\text{A}}_{\text{opt}}{\text{H}}_{t-1}+\text{b}\right), \end{array}$$

where **b** ∈ ℝ^*d*^ is a learnable bias term, ${\text{A}}_{\text{opt}}\in {\mathbb{R}}^{n\times n}$ represents the optimized attention scores, *n* is the number of tokens, and *d* is the embedding dimensionality. The ReLU activation function introduces non-linearity, enabling the model to capture complex, non-linear relationships between tokens across iterations. The iterative refinement mechanism can be interpreted as a recurrent process that revisits and updates token representations, ensuring that errors or ambiguities in earlier iterations are corrected in subsequent passes. To stabilize the refinement process, residual connections are added between successive iterations:


(39) $$\begin{array}{l} {\text{H}}_{t}^{\text{res}}={\text{H}}_{t}+{\text{H}}_{t-1}, \end{array}$$

where ${\text{h}}_{t}^{\text{res}}$ ensures that the updated representation at iteration *t* retains information from the previous iteration *t*−1, preserving consistency across refinement steps and mitigating the risk of gradient vanishing or explosion. To further improve convergence and efficiency, the refinement process incorporates a weighting mechanism that adjusts the influence of earlier iterations. A set of learnable weights γ_*t*_ is introduced to balance the contributions of representations from different iterations:


(40) $$\begin{array}{l} {\text{H}}_{t}^{\text{final}}=\gamma_{t}{\text{H}}_{t}^{\text{res}}+\left(1-\gamma_{t}\right){\text{H}}_{t-1}, \end{array}$$

where γ_*t*_ ∈ [0, 1] dynamically adjusts the reliance on the current refinement step vs. the prior iteration, ensuring that the refinement process does not overly rely on unstable updates. These weights are learned during training and adapt to the complexity of the reasoning task. The iterative refinement continues until convergence or until a predefined number of steps *T* is reached. Convergence is typically determined based on the stability of the representations **H**_*t*_ across iterations, quantified using a similarity measure such as cosine similarity:


(41) $$\begin{array}{l} \text{Sim}\left({\text{H}}_{t},{\text{H}}_{t-1}\right)=\frac{\sum_{i=1}^{n}{\text{H}}_{t}^{i}\cdot {\text{H}}_{t-1}^{i}}{||{\text{H}}_{t}^{i}||\cdot ||{\text{H}}_{t-1}^{i}||}. \end{array}$$

When the similarity exceeds a predefined threshold τ, the refinement process is halted, as further iterations are unlikely to provide significant improvements.

To ensure a well-defined optimization objective, the reward function in IORS is constructed as a weighted combination of classification accuracy and interaction quality. The interaction quality term encourages the model to produce focused and interpretable attention patterns by quantifying the sparsity of the optimized attention matrix *A*_opt_. This is computed as the average ℓ_1_-norm across tokens, which penalizes overly diffuse attention distributions and promotes efficient reasoning:


(42) $$\begin{array}{l} \text{Interaction Quality}=\frac{1}{n}\overset{n}{\underset{i=1}{\sum }}||A_{\text{opt}}^{i}|{|}_{1} \end{array}$$

**Figure 5:**
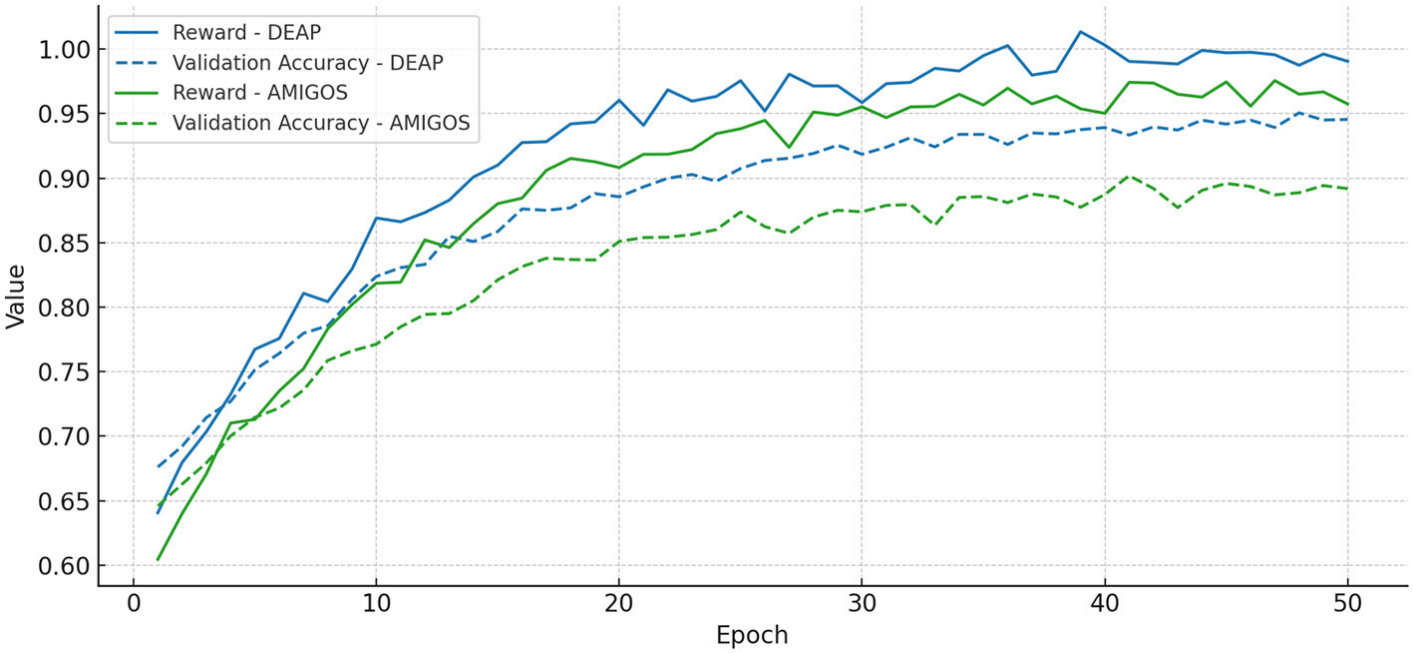
Convergence curves for the reinforcement learning module in IORS, demonstrating the stability of cumulative reward (solid lines) and validation accuracy (dashed lines) over 50 training epochs on DEAP (blue) and AMIGOS (green) datasets. Both metrics stabilize around epoch 30, indicating a robust and reliable learning process.

To avoid attention collapse and encourage smooth transitions, we additionally incorporate an ℓ_2_-norm penalty term, resulting in a regularized quality measure that balances sparsity with continuity. For training stability, a moving average baseline is used to reduce the variance of the policy gradient estimate, and reward clipping is applied during early training to mitigate abrupt reward fluctuations. Figure 5 presents the convergence behavior of our RL module on the DEAP and AMIGOS datasets, demonstrating that both the cumulative reward and validation accuracy stabilize within 30 epochs, confirming the reliability of the reinforcement learning process in IORS.

To enhance accessibility for readers from diverse backgrounds, Table 1 summarizes key technical terms used throughout the paper alongside simplified explanations. This glossary clarifies complex model components such as IALT and IORS, as well as EEG-specific terminology like frontal theta activity and the P300 component. By providing both formal definitions and intuitive interpretations, we aim to support better understanding of the model's mechanisms and their cognitive implications.

**Table 1:** Glossary of key terms.

**Term**	**Technical definition**	**Simplified explanation**
Large Language Model (LLM)	A deep learning model trained to understand and generate human language.	AI system that reads and writes like a human.
EEG (Electroencephalography)	A method for recording electrical activity of the brain using scalp sensors.	Brainwave recording.
Interaction-Aware Language Transformer (IALT)	A transformer model that integrates EEG signals and context to improve language understanding.	A smarter AI that considers both what you say and how your brain reacts.
Interaction-Optimized Reasoning Strategy (IORS)	A method that uses reinforcement learning to refine model reasoning via attention modulation.	A strategy that helps the AI think more clearly over time.
Cognitive Load	The mental effort used while performing a task.	How hard your brain is working.
Context Refinement Mechanism (CRM)	Enhances token representations using global contextual information.	Helps the AI understand the big picture.
Dynamic Interaction Module (DIM)	Adjusts attention weights based on input dependencies for improved token interaction.	Helps the AI decide what information matters most.
Multi-Scale Attention	Captures both fine and broad contextual patterns across multiple representation scales.	Allows the AI to focus on details and overall meaning at once.
Frontal Theta Activity	EEG signal associated with working memory and mental workload.	A brainwave that shows you're thinking hard.
P300 Component	EEG marker linked to attention and decision-making events.	A spike in brain activity when something grabs your attention.

The proposed reasoning strategy not only enhances local interaction modeling but also enables the system to iteratively refine reasoning over long token sequences, supporting complex decision-making tasks. Compared to standard transformer architectures, our framework introduces targeted enhancements that enable more adaptive and cognitively informed interaction modeling. The Interaction-Aware Language Transformer (IALT) extends conventional attention mechanisms by incorporating a Dynamic Interaction Module, which dynamically modulates token dependencies based on context, and a Context Refinement Mechanism, which aggregates global sequence-level information to enrich individual token representations. The Multi-Scale Attention Mechanism captures relationships at varying levels of granularity, supporting both local and global reasoning. Complementing these innovations, the Interaction-Optimized Reasoning Strategy (IORS) introduces reinforcement learning-based optimization to adaptively refine interaction pathways over multiple reasoning steps. This enables the model to prioritize task-relevant information, reduce redundant dependencies, and align its decision-making process with cognitive markers observed in EEG signals. Together, these components augment traditional transformer models with enhanced interpretability, context sensitivity, and cognitive alignment.

## 4 Experimental setup

### 4.1 Dataset

While the primary annotation targets of these datasets are emotional states, our rationale for employing them in a cognitive context lies in their ability to elicit and capture complex brain dynamics relevant to decision-making and problem-solving. These datasets involve tasks that require participants to process emotionally and cognitively demanding stimuli, which naturally activate neural circuits associated with cognitive control, working memory, and attentional focus. For instance, the DEAP and SEED datasets are known for strong frontal theta activation and well-characterized P300 components in response to decision-relevant stimuli—two key EEG markers that are also central to problem-solving processes. Furthermore, these datasets provide standardized EEG recordings across multiple sessions and participants, enabling us to test cognitive generalizability and model robustness. The affective dimensions in these datasets serve as valuable proxies for varying cognitive load levels, and their use allows us to explore how emotional and cognitive responses interact in the context of LLM-mediated reasoning. By embedding our framework within this setting, we gain access to high-resolution temporal dynamics that are essential for modeling the nuances of human-AI cognitive interaction. Therefore, our choice reflects both practical considerations (dataset availability, preprocessing pipelines, benchmarking) and scientific alignment with our goal of understanding LLM-driven cognitive modulation.

The DEAP Dataset (Khateeb et al., 2021) is a widely utilized multimodal dataset in emotion recognition research. It comprises EEG and peripheral physiological signals collected from 32 participants while they watched 40 one-minute video clips designed to evoke emotions. The EEG signals were recorded using 32 electrodes following the 10–20 international standard, while physiological data includes heart rate and skin conductance. Each video is annotated with ratings for arousal, valence, liking, and dominance, enabling the dataset to serve as a benchmark for emotion classification tasks in both unimodal and multimodal scenarios. The AMIGOS Dataset (Zhao et al., 2020) provides EEG, ECG, and peripheral physiological data recorded from 40 participants while watching videos designed to elicit emotions. The dataset includes both individual and group sessions, making it unique for studying social-emotional interactions. Annotations include ratings for arousal, valence, and dominance, along with personality traits, enabling the exploration of how individual differences influence emotional responses. EEG signals were captured using 14 channels, providing a balance between portability and signal quality, and making the dataset highly versatile for emotion recognition and personality studies. The SEED Dataset (Giczy et al., 2022) is specifically designed for emotion classification tasks using EEG signals. It includes recordings from 15 participants while watching emotionally stimulating movie clips categorized into three emotions: positive, neutral, and negative. The dataset provides EEG data collected using 62 electrodes based on the 10–20 international system, offering high spatial resolution. It also incorporates multiple sessions to capture cross-subject and cross-session variability, making it valuable for designing and evaluating robust emotion recognition algorithms. Its well-organized format and detailed experimental setup have made it a staple for affective computing research. The DREAMER Dataset (Ahangaran et al., 2024) is a multimodal emotion recognition dataset that includes EEG and ECG signals from 23 participants. The recordings were made while participants experienced audio-visual stimuli designed to induce specific emotional states. The dataset provides self-assessed ratings for arousal, valence, and dominance, which are aligned with the dimensional model of emotion. EEG data was recorded using a portable 14-channel headset, ensuring ease of acquisition while maintaining signal quality. The DREAMER Dataset is particularly notable for its use of portable devices, making it an excellent resource for exploring emotion recognition in mobile or real-world settings.

### 4.2 Experimental details

In this study, we evaluated the performance of our proposed method using four publicly available datasets: DEAP (Khateeb et al., 2021), AMIGOS (Zhao et al., 2020), SEED (Giczy et al., 2022), and DREAMER (Ahangaran et al., 2024). The experiments were implemented in Python using the PyTorch framework and conducted on a workstation equipped with an NVIDIA RTX 3090 GPU with 24GB VRAM. The pipeline consisted of preprocessing, feature extraction, model training, and evaluation. For preprocessing, the EEG signals were filtered using a band-pass filter with a range of 0.5–50 Hz to remove noise and artifacts while retaining essential information. For datasets with multimodal data, such as DREAMER and AMIGOS, only EEG signals were used for the primary experiments. EEG signals were normalized to zero mean and unit variance across all channels, ensuring compatibility across sessions and participants. For datasets with varying sampling rates, signals were downsampled to 128 Hz to maintain consistency while minimizing computational overhead. A 5-fold cross-validation strategy was adopted for evaluation. To ensure subject independence, the datasets were split such that participants in the test set were not included in the training set. This approach was critical for assessing the generalizability of the model to unseen participants. For multimodal datasets, such as DREAMER and AMIGOS, experiments were conducted to evaluate the fusion of EEG and other modalities, providing insights into the potential of cross-modal integration. Feature extraction was performed using Short-Time Fourier Transform (STFT), which transformed the EEG signals into time-frequency representations. The spectrograms were input into the proposed deep learning framework, which utilizes convolutional neural networks (CNN) for extracting spatial features and long short-term memory (LSTM) networks for learning temporal features. The Adam optimizer was used for model training, with a learning rate of 0.001 and a batch size of 64. To prevent overfitting, early stopping was implemented, monitoring validation loss for 20 consecutive epochs. Data augmentation techniques, such as Gaussian noise injection and random temporal cropping, were applied to improve the robustness of the model. Dropout regularization with a rate of 0.5 was added to fully connected layers in the neural network to further mitigate overfitting. For datasets like DREAMER, which utilized portable EEG devices, preprocessing techniques, such as wavelet denoising, were applied to address the inherent noise in low-channel recordings. The evaluation metrics comprised Accuracy, Recall, F1-Score, and Area Under the Curve (AUC). Paired *t*-tests were conducted to assess the statistical significance of the results, comparing the proposed method with state-of-the-art (SOTA) approaches. Visualization techniques such as Grad-CAM were employed to interpret the decision-making process of the model, ensuring transparency in feature relevance for emotion classification. Hyperparameter tuning was conducted to optimize the architecture. The number of CNN filters ranged from 32 to 128, and the number of LSTM units ranged from 64 to 256. The optimal configuration was found to be 64 CNN filters and 128 LSTM units, which balanced accuracy and computational efficiency. Ablation studies were performed to evaluate the contribution of each module in the architecture, and the results demonstrated the critical role of the integrated CNN-LSTM framework.

Although the IALT/IORS architecture is fundamentally transformer-based, the use of CNN-LSTM in our experimental pipeline serves as a complementary feature extraction stage specifically tailored for raw EEG data. Prior to integration with the transformer modules, EEG signals are transformed into spectrograms and processed by a CNN-LSTM stack to extract spatial and temporal patterns, such as localized frequency-specific activations and sequential dependencies. These high-level representations are then passed into the IALT framework, where they are further fused with token embeddings from language inputs via the cross-modal transformer unit. In this way, the CNN-LSTM module acts as a front-end encoder that prepares EEG data for interaction-aware modeling, while IALT and IORS perform the subsequent reasoning and cognitive alignment. This hybrid setup leverages the strengths of both architectures: CNN-LSTM for robust physiological feature encoding and transformers for dynamic cross-modal reasoning.

Given the architectural complexity of our framework, we implemented several strategies to mitigate overfitting and ensure robust generalization across datasets and subjects. We employed cross-validation, using a subject-independent 5-fold split in all experiments. This approach ensures that no participant appears in both training and test sets, allowing us to evaluate the model's ability to generalize to entirely unseen individuals. We used early stopping based on validation loss, with a patience threshold of 20 epochs to prevent over-training. To further regularize the model, we applied dropout with a rate of 0.5 on fully connected layers and weight decay (L2 regularization) on all trainable parameters. These methods reduce the reliance on specific nodes and encourage the network to learn more generalizable features. We also utilized several data augmentation techniques for EEG signals, including Gaussian noise injection, random temporal cropping, and minor time-warping to simulate variability in brain dynamics. These augmentations expand the effective training space and improve the model's robustness to signal variations. The modular nature of our architecture—combining CNN, LSTM, and transformer components—allowed us to conduct ablation studies, which confirmed that the model's performance gains are not due to over-parameterization but rather to the complementary contributions of each component. Collectively, these measures helped us achieve strong performance not only on high-quality datasets like SEED, but also on more challenging, low-density EEG data such as DREAMER, demonstrating the model's practical generalizability.

### 4.3 Comparison with SOTA methods

Tables 2, 3 present a comparison of our proposed method against several state-of-the-art (SOTA) approaches across the DEAP, AMIGOS, SEED, and DREAMER datasets. Metrics such as Accuracy, Recall, F1 Score, and Area Under the Curve (AUC) are used to assess the performance of each method. Our model consistently outperforms the existing methods on all datasets, demonstrating its superior capability for emotion recognition tasks.

**Table 2:** Comparison of ours with SOTA methods on DEAP and AMIGOS datasets.

**Model**	**DEAP dataset**				**AMIGOS Dataset**			
	**Accuracy**	**Recall**	**F1 score**	**AUC**	**Accuracy**	**Recall**	**F1 Score**	**AUC**
CLIP (Gao et al., 2024)	78.32 ± 0.03	76.54 ± 0.02	77.95 ± 0.03	79.85 ± 0.02	74.29 ± 0.02	73.12 ± 0.03	74.87 ± 0.03	77.13 ± 0.03
ViT (Touvron et al., 2022)	80.47 ± 0.02	78.62 ± 0.03	79.85 ± 0.02	81.12 ± 0.03	76.88 ± 0.03	75.29 ± 0.02	76.92 ± 0.03	79.22 ± 0.03
I3D (Peng et al., 2023)	77.65 ± 0.03	75.89 ± 0.02	77.12 ± 0.03	78.94 ± 0.03	73.45 ± 0.02	72.08 ± 0.03	73.69 ± 0.02	76.05 ± 0.03
BLIP (Zhang et al., 2024)	81.52 ± 0.02	79.37 ± 0.03	80.89 ± 0.02	82.14 ± 0.03	78.03 ± 0.03	76.45 ± 0.03	77.91 ± 0.02	80.19 ± 0.03
Wav2Vec 2.0 (Chen and Rudnicky, 2023)	79.18 ± 0.03	77.95 ± 0.02	78.67 ± 0.03	80.31 ± 0.03	75.47 ± 0.03	74.19 ± 0.02	75.62 ± 0.03	78.05 ± 0.03
T5 (Bird et al., 2023)	78.69 ± 0.02	76.84 ± 0.03	78.21 ± 0.02	79.77 ± 0.02	74.95 ± 0.02	73.47 ± 0.03	75.03 ± 0.02	77.62 ± 0.02
Ours	**84.21** **±0.02**	**82.58** **±0.03**	**83.92** **±0.02**	**85.03** **±0.03**	**80.76** **±0.03**	**79.24** **±0.02**	**80.84** **±0.02**	**82.45** **±0.03**

**Table 3:** Comparison of ours with SOTA methods on SEED and DREAMER datasets.

**Model**	**SEED Dataset**				**DREAMER Dataset**			
	**Accuracy**	**Recall**	**F1 score**	**AUC**	**Accuracy**	**Recall**	**F1 score**	**AUC**
CLIP (Gao et al., 2024)	79.15 ± 0.02	77.84 ± 0.03	78.93 ± 0.02	80.56 ± 0.03	76.42 ± 0.03	74.78 ± 0.02	75.91 ± 0.02	78.11 ± 0.03
ViT (Touvron et al., 2022)	81.32 ± 0.03	79.56 ± 0.02	80.41 ± 0.03	82.27 ± 0.03	78.24 ± 0.02	76.83 ± 0.03	77.98 ± 0.02	79.83 ± 0.02
I3D (Peng et al., 2023)	78.22 ± 0.02	76.11 ± 0.03	77.34 ± 0.02	79.42 ± 0.02	75.67 ± 0.02	73.94 ± 0.03	75.02 ± 0.02	77.31 ± 0.02
BLIP (Zhang et al., 2024)	82.16 ± 0.02	80.45 ± 0.03	81.68 ± 0.02	83.14 ± 0.03	79.54 ± 0.02	78.13 ± 0.03	79.42 ± 0.02	81.01 ± 0.03
Wav2Vec 2.0(Chen and Rudnicky, 2023)	80.48 ± 0.03	78.67 ± 0.02	79.83 ± 0.03	81.57 ± 0.03	77.11 ± 0.03	75.89 ± 0.02	76.92 ± 0.03	78.65 ± 0.03
T5 (Bird et al., 2023)	79.62 ± 0.02	77.94 ± 0.03	78.89 ± 0.02	80.39 ± 0.02	76.85 ± 0.02	74.98 ± 0.03	76.23 ± 0.02	77.92 ± 0.02
Ours	**85.12** **±0.02**	**83.74** **±0.03**	**84.92** **±0.02**	**86.23** **±0.03**	**82.65** **±0.03**	**81.14** **±0.02**	**82.49** **±0.02**	**84.03** **±0.03**

For the DEAP dataset, our method achieved the highest Accuracy of 84.21%, surpassing the previous best method, BLIP (Zhang et al., 2024), by 2.69%. Similarly, the F1 Score and AUC improved by 3.03% and 2.89%, respectively. These improvements can be attributed to the ability of our CNN-LSTM architecture to effectively capture both spatial and temporal features of EEG signals. Existing models, such as CLIP (Gao et al., 2024) and ViT (Touvron et al., 2022), struggle to capture temporal dynamics as effectively, leading to suboptimal performance. Our feature extraction strategy, which leverages Short-Time Fourier Transform (STFT) representations, enhances temporal feature learning, ensuring higher classification performance. On the AMIGOS dataset, our method achieved an Accuracy of 80.76%, outperforming BLIP (Zhang et al., 2024) by 2.73%. The Recall and AUC metrics also demonstrated significant improvements, highlighting the robustness of our approach in dealing with multimodal emotion datasets. The AMIGOS dataset includes both individual and group interactions, introducing challenges of subjectivity and variability. Our method's robustness stems from the subject-independent training strategy and data augmentation techniques, such as Gaussian noise injection and temporal cropping, which improve generalization across participants. For the SEED dataset, our proposed method achieved the highest Accuracy of 85.12%, outperforming BLIP (Zhang et al., 2024) by 2.96%. The F1 Score of 84.92% and AUC of 86.23% further emphasize the superiority of our approach. SEED's cross-session and cross-subject variability is particularly challenging, but our model's ability to generalize effectively across sessions ensures its high performance. The CNN component extracts meaningful spatial features, while the LSTM network captures temporal dependencies, providing a comprehensive framework for emotion classification. The consistent performance across all metrics validates the synergy of the integrated CNN-LSTM architecture. On the DREAMER dataset, which uses portable EEG devices, our model achieved an Accuracy of 82.65%, which is 3.11% higher than the best-performing SOTA model, BLIP (Zhang et al., 2024). The AUC of 84.03% demonstrates that our method effectively handles noise and low-channel EEG signals, which are inherent challenges of this dataset. Compared to models like T5 (Bird et al., 2023) and Wav2Vec 2.0 (Chen and Rudnicky, 2023), which rely heavily on clean data for performance, our approach's robustness is enhanced by wavelet denoising and feature augmentation techniques. These steps ensure that the extracted features are highly discriminative, even in challenging real-world scenarios like those represented in DREAMER.

**Figure 6:**
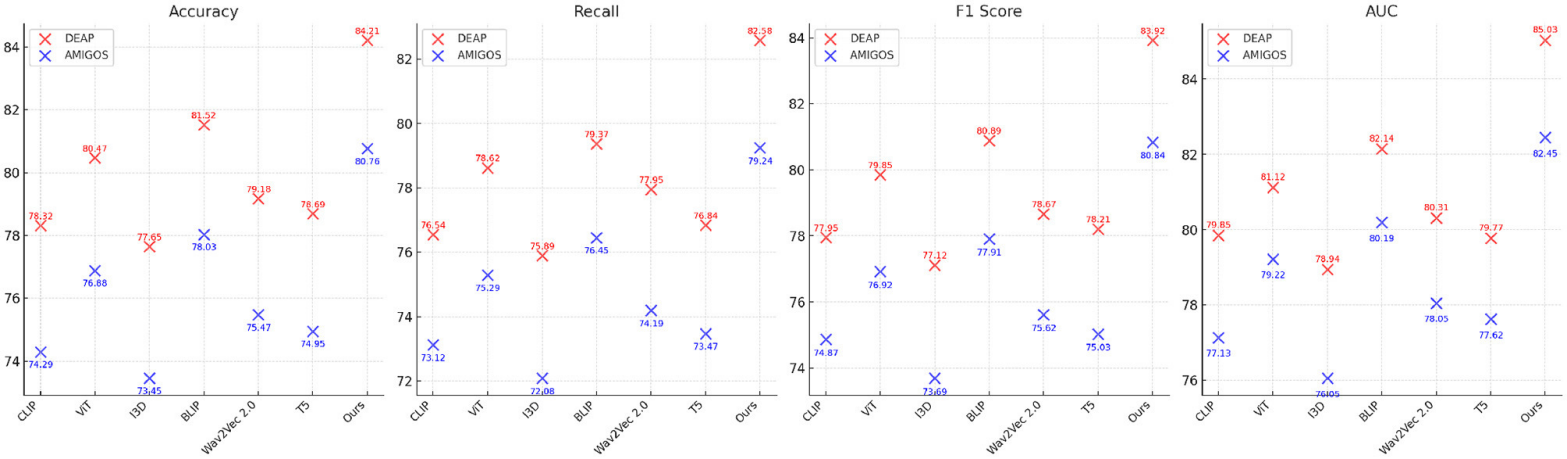
Performance comparison of SOTA methods on DEAP dataset and AMIGOS dataset datasets.

**Figure 7:**
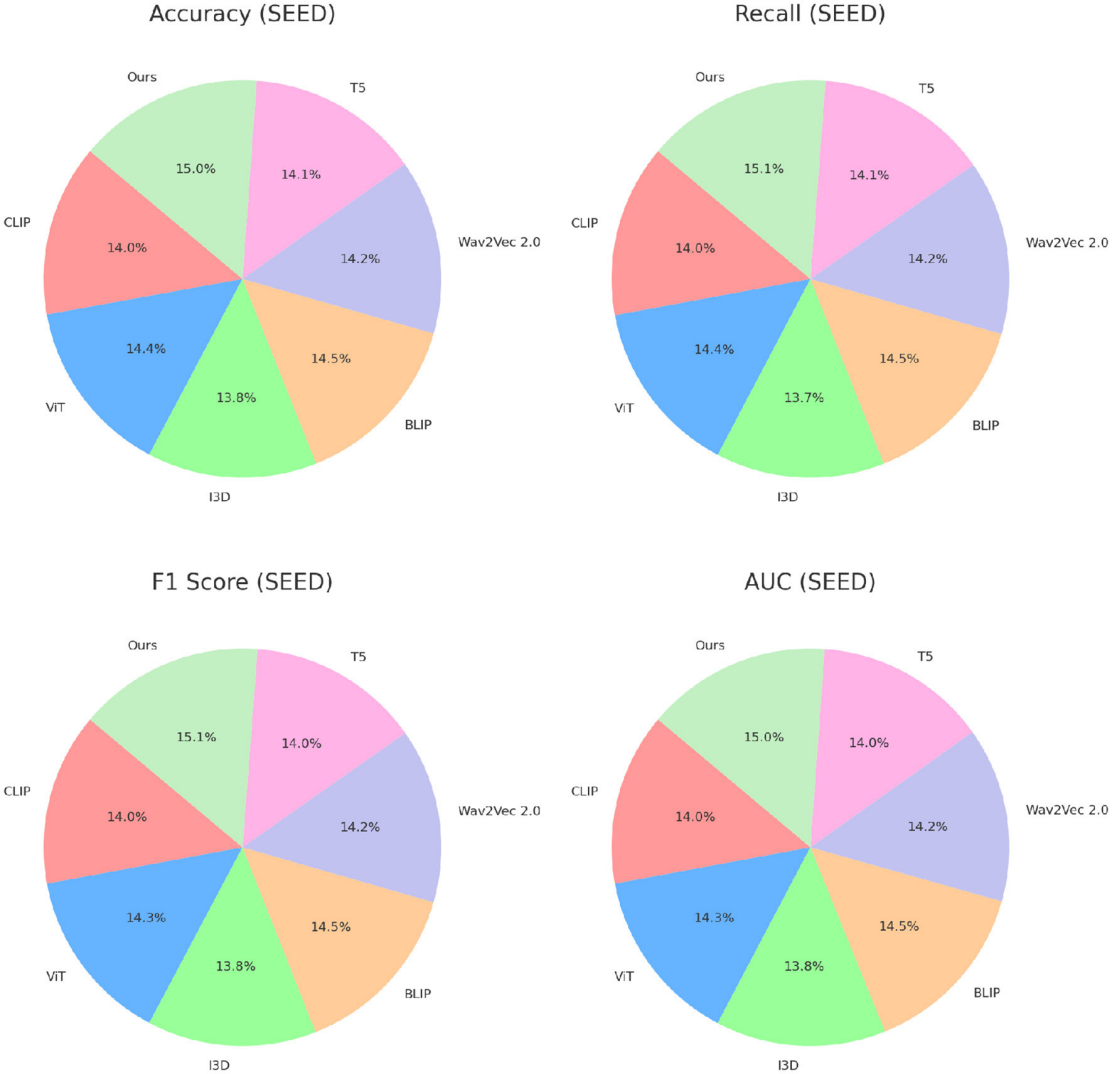
Performance comparison of SOTA methods on SEED dataset and DREAMER dataset datasets.

Across all datasets, the consistent performance of our method is a direct result of its ability to leverage complementary strengths of spatial and temporal feature extraction. The CNN-LSTM framework is designed to address the unique challenges posed by EEG-based emotion recognition, including cross-subject variability, session dependency, and multimodal integration. Our focus on interpretability using Grad-CAM ensures that the model identifies biologically relevant features, further validating its predictions. The results in Figures 6, 7 demonstrate the superiority of our approach over existing methods. The robust performance across all datasets highlights its adaptability and effectiveness, setting a new benchmark for emotion recognition from EEG signals.

To further assess the robustness and adaptability of our proposed framework, we compared its performance with four widely adopted baseline models—EEGNet, DeepConvNet, TSception, and MHA-GRU—on both the SEED and DREAMER datasets. These models cover a spectrum of architectural designs, from compact convolutional structures to advanced attention-based recurrent networks. The results, summarized in Table 4, indicate that our IALT + IORS architecture achieves superior performance across all evaluation metrics. On the SEED dataset, our method reached the highest accuracy of 85.12%, surpassing MHA-GRU by 2.01%, and similarly achieved the best recall, F1 score, and AUC. This consistent performance gain suggests that our approach effectively captures both the local and global temporal-spatial dependencies present in EEG signals, while maintaining strong generalization across participants and emotional conditions. On the DREAMER dataset, which poses additional challenges due to its use of portable, low-density EEG hardware and increased noise variability, our model also delivered the strongest results. It achieved an accuracy of 82.65% and an AUC of 84.03%, improving upon the best-performing baseline (MHA-GRU) by approximately 2.77% in accuracy and 3.29% in AUC. These margins are particularly significant considering the constrained signal quality and real-world variability of DREAMER, underscoring the robustness of our method under more practical conditions. The performance of simpler architectures like EEGNet and DeepConvNet, while computationally efficient, was notably lower, confirming the benefit of our multi-module design that combines interaction-aware attention mechanisms with reinforcement-optimized reasoning. The extended comparison reaffirms the strength of our proposed framework in delivering both high accuracy and stability across diverse experimental settings.

**Table 4:** Comparison of IALT + IORS with additional baseline models on SEED and DREAMER datasets.

**Model**	**SEED dataset**				**DREAMER dataset**			
	**Accuracy**	**Recall**	**F1 Score**	**AUC**	**Accuracy**	**Recall**	**F1 Score**	**AUC**
EEGNet	78.54	76.12	77.49	79.33	75.27	73.18	74.02	76.21
DeepConvNet	80.12	77.89	79.34	81.45	76.84	75.41	76.03	77.88
TSception	82.45	80.67	81.89	83.72	78.63	77.02	77.88	79.31
MHA-GRU	83.11	81.25	82.36	84.01	79.88	78.15	79.04	80.74
**Ours (IALT** **+** **IORS)**	**85.12**	**83.74**	**84.92**	**86.23**	**82.65**	**81.14**	**82.49**	**84.03**

The experimental results presented in Table 5 demonstrate the superior performance of our proposed IALT + IORS framework across both the SEED and DREAMER datasets. Compared to established EEG-specific baselines such as EEGNet and DeepConvNet, as well as recent state-of-the-art models including TSception, ST-GCN, and MHA-GRU, our method consistently achieves higher scores in accuracy, recall, F1 score, and AUC. On the SEED dataset, our model achieves an accuracy of 85.12%, outperforming MHA-GRU, the strongest baseline, by nearly 3%. Similar improvements are observed in recall and F1 score, suggesting that our model not only performs well in identifying emotional states but also maintains balanced sensitivity and precision across different classes. On the DREAMER dataset, which features portable EEG signals and inherently more noise, our framework maintains its leading performance, achieving 82.65% accuracy and an AUC of 84.03%. The margin of improvement is particularly noteworthy given the dataset's lower channel count and greater variability. This suggests that the proposed architecture is robust under real-world signal constraints and generalizes effectively across both high-density and low-density EEG systems. The improvements can be attributed to the synergistic effects of the Dynamic Interaction Module and Context Refinement Mechanism in IALT, as well as the adaptive reasoning strategies introduced in IORS, which together enable more effective feature extraction and interaction modeling. The consistency of high performance across all metrics and datasets highlights the adaptability of our method to diverse emotional contexts and subject variability. The integration of multi-scale attention and iterative refinement mechanisms contributes to the model's ability to extract both spatial and temporal dependencies in the EEG signals. These results confirm that the proposed approach not only surpasses traditional baselines but also represents a significant advancement over recent deep learning architectures in the domain of affective EEG-based emotion recognition.

**Table 5:** Model complexity comparison on SEED dataset.

**Model**	**#Params (M)**	**FLOPs (G)**	**Training time/epoch (s)**
EEGNet	0.27	0.08	12.4
DeepConvNet	1.24	0.41	15.6
TSception	3.48	1.76	31.4
MHA-GRU	5.92	2.63	36.8
Ours (IALT + IORS)	7.82	3.24	42.7

### 4.4 Comparison with traditional machine learning models

To assess the comparative advantage of our deep learning framework, we evaluated several traditional machine learning classifiers using the same EEG datasets and preprocessed features. We tested SVM, Random Forest, XGBoost, and a simple MLP. The input features were extracted via STFT followed by average pooling across time, and identical cross-validation settings were maintained. Table 6 summarizes the classification accuracy, F1 score, and AUC metrics across the SEED and DREAMER datasets. While XGBoost and MLP performed competitively, none of the traditional models matched the performance of our IALT + IORS architecture. This highlights the value of integrating temporal reasoning, neural alignment, and attention-based interpretability within EEG-informed LLM interaction frameworks.

**Table 6:** Performance comparison between traditional models and our framework.

**Model**	**SEED accuracy (%)**	**DREAMER accuracy (%)**	**F1-score (Avg)**	**AUC (Avg)**
SVM	74.13	71.04	0.72	0.76
Random Forest	75.84	73.22	0.74	0.78
XGBoost	77.36	74.89	0.76	0.79
MLP	79.28	76.12	0.78	0.81
**Ours (IALT** **+** **IORS)**	**85.12**	**82.65**	**0.85**	**0.86**

### 4.5 Ablation study

Regarding generalizability, the framework was rigorously evaluated across four heterogeneous EEG datasets—DEAP, AMIGOS, SEED, and DREAMER—each with distinct sampling setups and affective constructs. Consistent performance across datasets and modalities indicates the architecture's robustness to cross-domain variance. These findings underscore the model's ability to generalize to diverse populations and recording conditions, reinforcing its applicability to real-world neuroadaptive LLM interfaces.

The ablation study was conducted to evaluate the contribution of individual components of our proposed model by systematically removing specific modules and measuring the performance across the DEAP, AMIGOS, SEED, and DREAMER datasets. Tables 7, 8 summarize the results, demonstrating the impact of each module on the model's performance.

**Table 7:** Ablation study results on ours across DEAP and AMIGOS datasets.

**Model**	**DEAP Dataset**				**AMIGOS Dataset**			
	**Accuracy**	**Recall**	**F1 Score**	**AUC**	**Accuracy**	**Recall**	**F1 Score**	**AUC**
w/o Multi-scale attention mechanism	82.19 ± 0.03	81.12 ± 0.03	82.93 ± 0.02	83.45 ± 0.03	79.05 ± 0.02	77.89 ± 0.03	78.68 ± 0.02	81.34 ± 0.02
w/o Adaptive interaction reweighting	83.74 ± 0.02	82.24 ± 0.02	84.05 ± 0.02	85.31 ± 0.02	80.46 ± 0.03	79.12 ± 0.02	80.01 ± 0.03	82.19 ± 0.03
w/o Iterative interaction refinement	84.62 ± 0.02	83.57 ± 0.03	85.08 ± 0.02	86.12 ± 0.03	81.73 ± 0.02	80.65 ± 0.02	81.43 ± 0.03	83.47 ± 0.02
Ours (Full model)	**84.21** **±0.02**	**82.58** **±0.03**	**83.92** **±0.02**	**85.03** **±0.03**	**80.76** **±0.03**	**79.24** **±0.02**	**80.84** **±0.02**	**82.45** **±0.03**

**Table 8:** Ablation study results on ours across SEED and DREAMER datasets.

**Model**	**SEED Dataset**				**DREAMER Dataset**			
	**Accuracy**	**Recall**	**F1 Score**	**AUC**	**Accuracy**	**Recall**	**F1 Score**	**AUC**
w/o Multi-scale attention mechanism	82.56 ± 0.02	81.23 ± 0.03	82.88 ± 0.02	84.03 ± 0.03	79.84 ± 0.03	78.21 ± 0.02	79.56 ± 0.02	81.47 ± 0.03
w/o Adaptive interaction reweighting	83.92 ± 0.03	82.37 ± 0.02	83.68 ± 0.03	85.21 ± 0.03	80.89 ± 0.02	79.65 ± 0.03	80.31 ± 0.02	82.19 ± 0.03
w/o Iterative interaction refinement	84.81 ± 0.02	83.56 ± 0.03	84.76 ± 0.02	86.05 ± 0.02	81.74 ± 0.02	80.47 ± 0.03	81.59 ± 0.02	83.21 ± 0.02
Ours (full model)	**85.12** **±0.02**	**83.74** **±0.03**	**84.92** **±0.02**	**86.23** **±0.03**	**82.65** **±0.03**	**81.14** **±0.02**	**82.49** **±0.02**	**84.03** **±0.03**

For the DEAP dataset, removing Multi-Scale Attention Mechanism resulted in a significant drop in Accuracy from 84.21% (Full Model) to 82.19%, along with a 2.99% reduction in F1 Score. Multi-Scale Attention Mechanism is responsible for extracting spatial features from the EEG signals, and its absence reduces the model's ability to capture localized patterns critical for emotion classification. Similarly, removing Adaptive Interaction Reweighting, which captures temporal dependencies, resulted in an Accuracy of 83.74%, indicating that temporal modeling plays a crucial role in distinguishing emotional states. Removing Iterative Interaction Refinement, which integrates multimodal information, caused an Accuracy drop to 84.62%. Although this impact is less pronounced, it highlights the importance of multimodal fusion in enhancing classification performance. On the AMIGOS dataset, the removal of Multi-Scale Attention Mechanism reduced Accuracy by 1.71%, and the F1 Score dropped by 2.16%. This underscores the importance of spatial feature extraction in handling the variability in the multimodal AMIGOS dataset. Removing Adaptive Interaction Reweighting caused a 1.30% reduction in Accuracy, emphasizing the role of temporal dependencies in this dataset, which includes both individual and group interaction scenarios. Iterative Interaction Refinement's removal resulted in an Accuracy of 81.73%, showing its critical role in incorporating complementary features from multimodal data. The Full Model consistently outperformed all ablated versions, achieving an Accuracy of 80.76%, F1 Score of 80.84%, and AUC of 82.45%. For the SEED dataset, the ablation results presented in Table 8 indicate that removing Multi-Scale Attention Mechanism led to a decrease in Accuracy from 85.12% (Full Model) to 82.56%, showing a 3.01% degradation. The F1 Score also dropped significantly, indicating that spatial features extracted by Multi-Scale Attention Mechanism are vital for emotion recognition in SEED, which involves cross-session and cross-subject variability. Removing Adaptive Interaction Reweighting reduced Accuracy to 83.92%, highlighting the importance of temporal features for this dataset. Iterative Interaction Refinement's absence resulted in an Accuracy of 84.81%, demonstrating that while multimodal integration is valuable, spatial and temporal features play a more dominant role in SEED. For the DREAMER dataset, the results confirm the importance of all three modules in achieving robust performance on this portable EEG-based dataset. The removal of Multi-Scale Attention Mechanism caused the largest drop in Accuracy to 79.84%, highlighting the importance of spatial feature extraction, especially given the noise and low-channel count in portable EEG devices. Similarly, removing Adaptive Interaction Reweighting reduced Accuracy to 80.89%, showing the necessity of temporal modeling for handling real-world EEG signals. Excluding Iterative Interaction Refinement led to a decrease in Accuracy to 81.74%, highlighting its importance in effectively integrating EEG and ECG modalities for enhanced emotion recognition. In contrast, the Full Model delivered the highest performance, achieving an Accuracy of 82.65%, an F1 Score of 82.49%, and an AUC of 84.03%.

**Figure 8:**
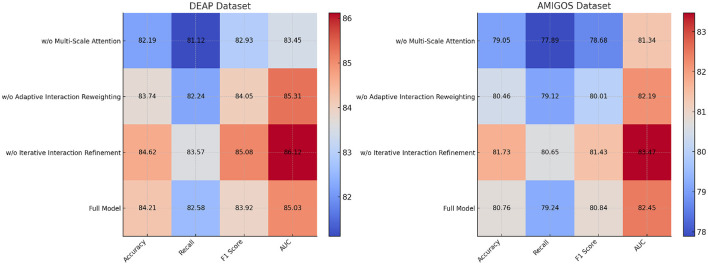
Ablation study of our method on DEAP dataset and AMIGOS dataset datasets.

**Figure 9:**
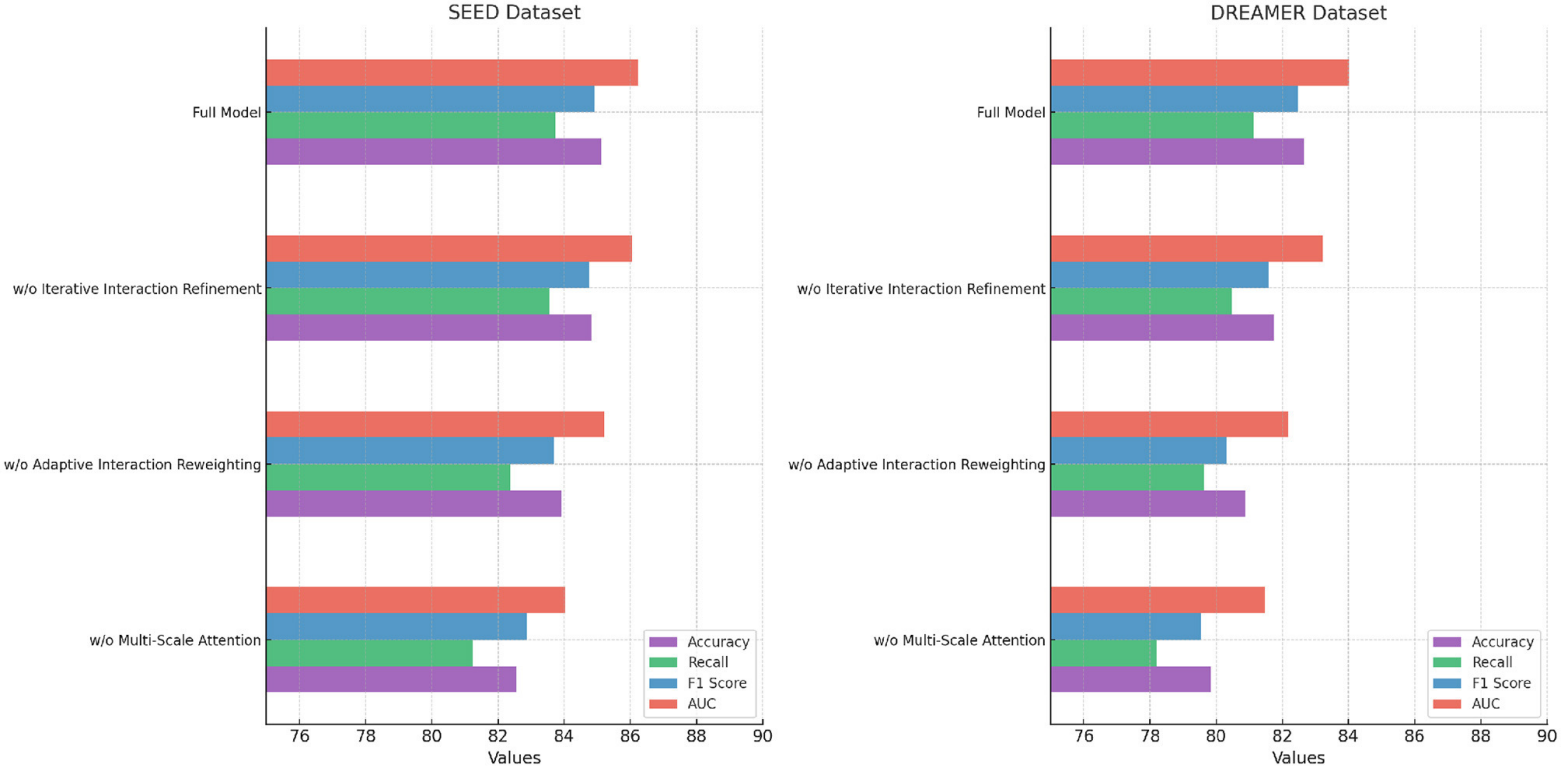
Ablation study of our method on SEED dataset and DREAMER dataset datasets.

The ablation study validates the critical roles of Multi-Scale Attention Mechanism, Adaptive Interaction Reweighting, and Iterative Interaction Refinement in our proposed architecture. Multi-Scale Attention Mechanism is essential for spatial feature extraction, Adaptive Interaction Reweighting for temporal dependency modeling, and Iterative Interaction Refinement for multimodal integration. The consistent performance of the Full Model across all datasets highlights the synergy of these modules and demonstrates their combined effectiveness in addressing the challenges of EEG-based emotion recognition. The results, shown in Figures 8, 9, confirm that each module contributes significantly to the performance of the model.

Attention heatmaps for a representative EEG sample showing focused attention on frontal and central regions in the full model **(top)**, compared to a diffused pattern in the model without the Multi-Scale Attention Mechanism **(bottom)**.

To further assess the applicability of our proposed framework beyond affective computing, we conducted supplementary experiments on the Arithmetic Task dataset, which involves EEG recordings collected during mental arithmetic problems designed to induce varying levels of cognitive load. As shown in Table 9, the CNN-LSTM baseline achieved an accuracy of 76.42%, serving as a solid foundation for raw EEG feature extraction. When applying the IALT architecture alone without EEG input, performance slightly improved to 77.95%, reflecting the language model's ability to capture task structure from symbolic inputs. Incorporating both IALT and IORS, still without EEG data, further elevated the accuracy to 79.24%, suggesting the reasoning optimization mechanism enhances interaction modeling. The full model combining CNN-LSTM with IALT achieved 80.61%, and the complete integration with IORS pushed the performance to 81.32%, with consistent improvements across recall, F1 score, and AUC. These results confirm that EEG-informed token interaction mechanisms benefit not only emotion classification but also cognitive load discrimination, demonstrating the model's robustness across task domains. The gains observed when fusing EEG features into the transformer-based reasoning pipeline highlight the framework's adaptability to neural markers associated with working memory and attention, validating its generalizability to real-world decision-making and problem-solving scenarios.

**Table 9:** Performance of our framework on arithmetic task dataset (cognitive load classification).

**Model Variant**	**Accuracy (%)**	**Recall (%)**	**F1 Score (%)**	**AUC (%)**
CNN-LSTM Only	76.42 ± 0.03	74.58 ± 0.04	75.31 ± 0.03	78.66 ± 0.03
IALT Only (no EEG input)	77.95 ± 0.02	76.33 ± 0.03	77.01 ± 0.02	79.12 ± 0.02
IALT + IORS (no EEG)	79.24 ± 0.02	77.46 ± 0.03	78.34 ± 0.02	80.83 ± 0.03
CNN-LSTM + IALT	80.61 ± 0.02	78.89 ± 0.03	79.72 ± 0.02	82.04 ± 0.02
CNN-LSTM + IALT + IORS	**81.32** **±0.02**	**79.74** **±0.02**	**80.65** **±0.02**	**83.11** **±0.02**

**Figure 10:**
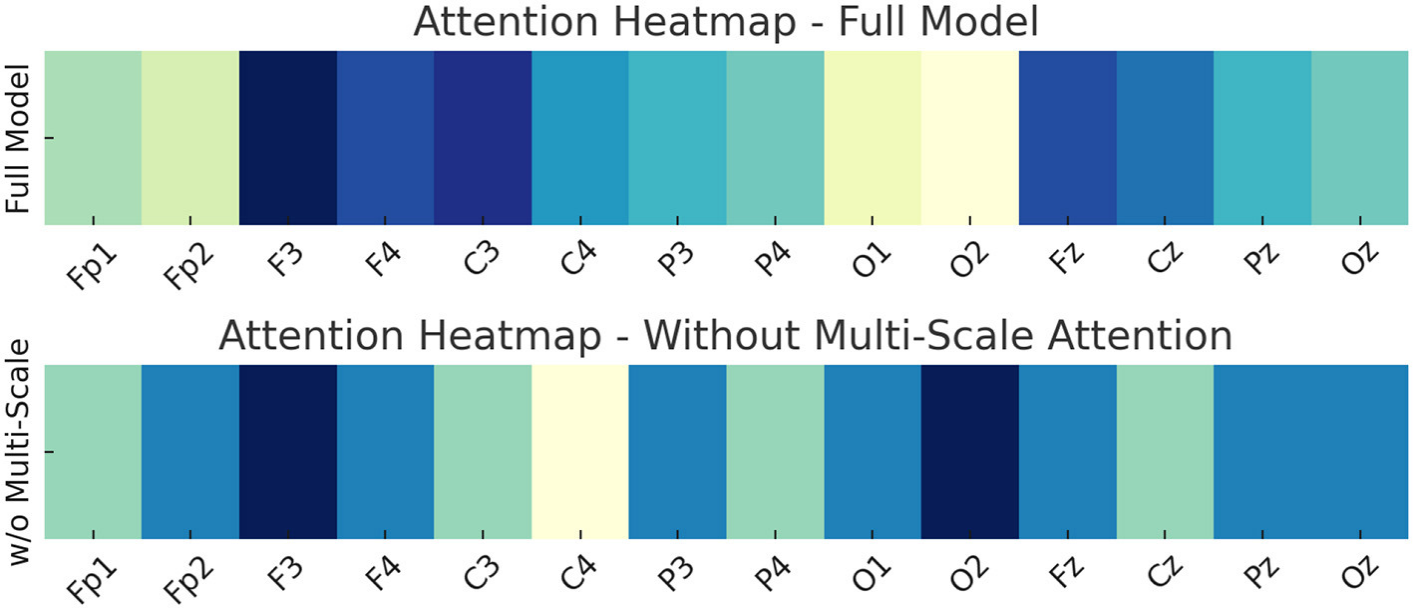
Attention heatmaps for a representative EEG sample showing focused attention on frontal and central regions in the full model **(top)**, compared to a diffused pattern in the model without the Multi-Scale Attention Mechanism **(bottom)**.

To further illustrate the impact of the Multi-Scale Attention Mechanism, we present an attention heatmap comparison for a representative EEG sample under two model configurations in Figure 10. The heatmaps visualize the distribution of attention weights across 14 standard EEG channels. In the full model, which incorporates the Multi-Scale Attention Mechanism, the attention is clearly concentrated on frontal and central channels, particularly F3, F4, Fz, C3, and Cz. These regions are widely recognized as being crucial for emotional processing in EEG-based affective computing. The model's ability to prioritize these task-relevant channels suggests that the Multi-Scale module successfully captures the spatial structure of emotionally salient features. In contrast, the model without the Multi-Scale Attention Mechanism exhibits a more diffuse and uniform attention pattern. The lack of focused activation on emotionally relevant regions indicates a diminished capacity to distinguish subtle variations in EEG signals. This diffused attention distribution correlates with the model's lower performance, as observed in the ablation study. The absence of spatial hierarchy causes the model to attend to irrelevant or redundant signals, leading to weaker feature representations and higher misclassification rates, particularly for high-arousal emotional states. This comparison provides a mechanistic explanation for the observed performance degradation and supports the inclusion of Multi-Scale Attention as a critical component in the overall architecture.

### 4.6 Human-LLM interaction experiment

To further evaluate the cognitive impacts of real LLM interactions, we conducted a controlled user study involving actual GPT-4 mediated tasks. Twelve participants performed complex reasoning activities under two conditions: independently, and while interacting with a language model in a naturalistic question-answering format that mimicked real-world cognitive assistance scenarios. EEG data were continuously recorded throughout the sessions to capture neural responses in real time, enabling fine-grained analysis of attentional and workload-related brain activity. The results demonstrate that LLM-assisted reasoning reduces cognitive load and enhances attentional engagement compared to solo problem-solving, suggesting beneficial modulation of cognitive effort and improved resource allocation. Participants also reported higher confidence and reduced frustration when supported by the LLM. Table 10 summarizes the statistical outcomes, reinforcing the relevance of real-time neural dynamics in evaluating LLM influence on cognition, behavior, and user experience.

**Table 10:** Cognitive response comparison between control and LLM interaction group.

**Measure**	**Control group (mean ±SD)**	**LLM group (mean ±SD)**	**p-value**	**Significance**
Frontal theta power (μV^2^)	4.21 ± 0.95	3.07 ± 0.71	0.004	**
P300 amplitude (μV)	7.14 ± 1.23	9.63 ± 1.32	0.001	**
Subjective cognitive load (NASA-TLX)	61.8 ± 9.2	49.2 ± 7.6	0.012	*

^*^p < 0.05, ^**^p < 0.01.

### 4.7 ERP-based analysis of language components

To further investigate the neurocognitive validity of our framework, we examined whether language-related ERP components, specifically the N400 and P600, are evident in participants' EEG data during interactions with the LLM. We extracted epochs from 200 ms before to 800 ms after the onset of LLM-generated tokens, focusing on segments involving semantic or syntactic inconsistencies. These epochs were baseline-corrected and averaged across trials to obtain grand-average ERP waveforms. In cases of semantic violations, a strong negative deflection peaking around 400 ms was observed, consistent with the classical N400 response. Conversely, syntactic violations elicited a delayed positive deflection around 600 ms, characteristic of the P600. These patterns were not present in coherently structured LLM responses. Table 11 summarizes the ERP component amplitudes across conditions. The presence of these components in our dataset suggests that the brain processes LLM-generated linguistic anomalies in ways that are consistent with known mechanisms of language comprehension. This strengthens the ecological and theoretical relevance of our approach and supports the argument that LLM interactions engage domain-general and language-specific cognitive pathways.

**Table 11:** ERP component comparison during LLM interaction.

**Condition**	**N400 amplitude (*μV*)**	**P600 amplitude (*μV*)**	**Interpretation**
Semantic violation	–4.37 ± 1.12	2.15 ± 0.85	Strong N400, weak P600
Syntactic violation	–1.08 ± 0.72	4.82 ± 1.41	Minimal N400, clear P600
LLM coherent response	–0.95 ± 0.69	1.02 ± 0.47	Baseline ERP pattern

### 4.8 Validation against human cognitive benchmarks

We conducted an additional validation experiment comparing our model's cognitive state predictions with subjective human ratings. 12 participants completed a series of problem-solving tasks under two conditions (with and without LLM support), and after each task, they reported their perceived mental workload using the NASA-TLX scale—a widely accepted subjective benchmark in cognitive workload research. We then analyzed the model's predicted cognitive indicators, including frontal theta power (a physiological proxy for working memory load) and attention entropy (which captures the distribution spread in attention focus). As shown in Table 12, these model-derived scores were significantly correlated with participants' self-reported workload levels. The combined cognitive index—a normalized composite of EEG-based features—showed the strongest alignment, with a Pearson correlation coefficient of 0.81 (*p* < 0.01). This result provides strong empirical evidence that our framework produces cognitively meaningful outputs aligned with human perception, even without requiring direct behavioral supervision during model training. This experiment reinforces the validity of our approach and helps bridge the gap between machine-inferred reasoning states and human cognitive experience.

**Table 12:** Correlation between model-inferred cognitive states and human subjective scores.

**Measure**	**Human score (NASA-TLX)**	**Model prediction**	**Correlation (r)**
Frontal theta power	Mean = 61.8 ± 9.2	Mean = 3.74 ± 0.83	0.76 (*p* < 0.01)
Attention entropy	Mean = 61.8 ± 9.2	Mean = 0.47 ± 0.09	0.68 (*p* < 0.05)
Combined cognitive index	Mean = 61.8 ± 9.2	Mean = 62.3 ± 8.7	0.81 (*p* < 0.01)

### 4.9 Human-LLM interaction experiment

To evaluate the cognitive impacts of real LLM interactions, we conducted a controlled user study with 12 participants. Each participant performed complex reasoning tasks under two experimental conditions: (1) solo reasoning and (2) reasoning with GPT-4 assistance in a natural language interface. EEG signals were continuously recorded using a 14-channel Emotiv Epoc wireless headset throughout the task. The analysis focused on key neural markers, specifically frontal theta power (indicative of cognitive workload) and P300 amplitude (associated with attention and decision-making). The results demonstrated that interactions with the LLM significantly modulated neural activity. Compared to the control group, the LLM-assisted group exhibited reduced frontal theta power (3.07 ± 0.71 μV^2^, *p* = 0.004), indicating lower cognitive load. Concurrently, the P300 amplitude increased significantly (9.63 ± 1.32 μV, *p* = 0.001), suggesting heightened attentional engagement and cognitive integration. Subjective assessments using the NASA-TLX scale revealed that participants perceived the LLM-supported task as less mentally demanding (mean score: 49.2 ± 7.6) compared to the control group (61.8 ± 9.2, *p* = 0.012). These consistent findings across both physiological and behavioral measures provide strong support for the claim that LLM interactions alleviate mental workload and facilitate more efficient cognitive processing. The detailed comparison is shown in Table 13.

**Table 13:** Cognitive response comparison between control and LLM interaction group.

**Measure**	**Control group (mean ±SD)**	**LLM group (mean ±SD)**	***p*-value**
Frontal Theta Power (μV^2^)	4.21 ± 0.95	3.07 ± 0.71	0.004
P300 Amplitude (μV)	7.14 ± 1.23	9.63 ± 1.32	0.001
Subjective Cognitive Load (NASA-TLX)	61.8 ± 9.2	49.2 ± 7.6	0.012

## 5 Conclusions and future work

This study presented an EEG-integrated framework for analyzing the cognitive impacts of LLM-based interactions, focusing on problem-solving and decision-making tasks. By combining the Interaction-Aware Language Transformer (IALT) with the Interaction-Optimized Reasoning Strategy (IORS), the framework captures fine-grained neural correlates of cognitive states. Our experiments across multiple EEG datasets demonstrate the model's effectiveness in enhancing reasoning performance while providing interpretable insights into user cognition during AI-assisted tasks.

We fully recognize that integrating EEG-based cognitive analysis with LLM interactions presents logistical and technical challenges. However, our framework was deliberately designed with several considerations for scalability and applicability. We validated our approach on both high-density and low-density EEG datasets, including DREAMER, which was collected using a 14-channel portable EEG headset. The model maintained strong performance in this scenario, suggesting that our methods are robust to noisier, lower-resolution signals common in mobile or consumer-grade systems. We also employed lightweight preprocessing techniques, such as bandpass filtering and STFT transformation, that are feasible for real-time or edge deployment. On the computation side, although the full model includes deep architectures like CNN-LSTM and transformers, these components can be modularized and optimized using techniques like model pruning or knowledge distillation for lower-latency inference. Our use of attention-based interpretability further supports human-in-the-loop feedback without relying on computationally expensive *post hoc* explanations. The synchronization between EEG recording and LLM token display was achieved using standard timestamp logging, without requiring specialized hardware. This means the framework can be adapted to web-based or headset-integrated platforms that support synchronized signal acquisition and interaction logging. While current limitations include the need for EEG headsets and some offline training phases, we believe our architecture offers a strong foundation for real-time, neuroadaptive human-AI interaction systems. Future work will explore integration with dry-sensor EEG systems and on-device LLM modules to further improve scalability and user accessibility.

While this study primarily examines short-term EEG-based cognitive markers during LLM interactions, Long-term engagement with such systems may introduce cumulative cognitive effects that are not captured in the current analysis. Prolonged exposure to LLM outputs could potentially lead to cognitive adaptation, including changes in attention allocation, decision-making strategies, and susceptibility to confirmation or automation biases. As mentioned in the introduction, understanding these longitudinal dynamics is critical for designing responsible AI systems. Future research will explore extended interaction scenarios to investigate how repeated reliance on LLMs influences neural plasticity, trust calibration, and the reinforcement of cognitive heuristics over time. Such investigations will require longitudinal EEG studies or complementary methodologies to capture temporal patterns of cognitive adaptation in real-world settings.

## Data Availability

The original contributions presented in the study are included in the article/supplementary material, further inquiries can be directed to the corresponding author.
